# A benchmark and an algorithm for detecting germline transposon insertions and measuring *de novo* transposon insertion frequencies

**DOI:** 10.1093/nar/gkab010

**Published:** 2021-01-28

**Authors:** Tianxiong Yu, Xiao Huang, Shengqian Dou, Xiaolu Tang, Shiqi Luo, William E Theurkauf, Jian Lu, Zhiping Weng

**Affiliations:** Department of Thoracic Surgery, Clinical Translational Research Center, Shanghai Pulmonary Hospital, The School of Life Sciences and Technology, Tongji University, Shanghai 200092, China; Program in Bioinformatics and Integrative Biology, University of Massachusetts Medical School, Worcester, MA 01605, USA; Department of Thoracic Surgery, Clinical Translational Research Center, Shanghai Pulmonary Hospital, The School of Life Sciences and Technology, Tongji University, Shanghai 200092, China; State Key Laboratory of Protein and Plant Gene Research, Center for Bioinformatics, School of Life Sciences and Peking-Tsinghua Center for Life Sciences, Peking University, Beijing 100871, China; State Key Laboratory of Protein and Plant Gene Research, Center for Bioinformatics, School of Life Sciences and Peking-Tsinghua Center for Life Sciences, Peking University, Beijing 100871, China; State Key Laboratory of Protein and Plant Gene Research, Center for Bioinformatics, School of Life Sciences and Peking-Tsinghua Center for Life Sciences, Peking University, Beijing 100871, China; Program in Molecular Medicine, University of Massachusetts Medical School, Worcester, MA 01605, USA; State Key Laboratory of Protein and Plant Gene Research, Center for Bioinformatics, School of Life Sciences and Peking-Tsinghua Center for Life Sciences, Peking University, Beijing 100871, China; Department of Thoracic Surgery, Clinical Translational Research Center, Shanghai Pulmonary Hospital, The School of Life Sciences and Technology, Tongji University, Shanghai 200092, China; Program in Bioinformatics and Integrative Biology, University of Massachusetts Medical School, Worcester, MA 01605, USA

## Abstract

Transposons are genomic parasites, and their new insertions can cause instability and spur the evolution of their host genomes. Rapid accumulation of short-read whole-genome sequencing data provides a great opportunity for studying new transposon insertions and their impacts on the host genome. Although many algorithms are available for detecting transposon insertions, the task remains challenging and existing tools are not designed for identifying *de novo* insertions. Here, we present a new benchmark fly dataset based on PacBio long-read sequencing and a new method TEMP2 for detecting germline insertions and measuring *de novo* ‘singleton’ insertion frequencies in eukaryotic genomes. TEMP2 achieves high sensitivity and precision for detecting germline insertions when compared with existing tools using both simulated data in fly and experimental data in fly and human. Furthermore, TEMP2 can accurately assess the frequencies of *de novo* transposon insertions even with high levels of chimeric reads in simulated datasets; such chimeric reads often occur during the construction of short-read sequencing libraries. By applying TEMP2 to published data on hybrid dysgenic flies inflicted by de-repressed *P-elements*, we confirmed the continuous new insertions of *P-elements* in dysgenic offspring before they regain piRNAs for *P-element* repression. TEMP2 is freely available at Github: https://github.com/weng-lab/TEMP2.

## INTRODUCTION

First discovered in the maze, transposons are mobile genetic elements that constitute up to 85% of metazoan genomes (1). Although transposons are often regarded as junk DNA, they play important roles in genomic variation and evolution (2–4). The movement of transposons often leads to genome instability and diseases, including infertility and cancer (5–8). Therefore, it is of great interest to annotate transposon insertion profiles in different cell types, during development, in disease states, or across individuals in a population. However, accurate detection of transposon insertions remains a challenge due to the highly repetitive sequences of transposons. Furthermore, short-read sequencing libraries contain chimeric reads, which are difficult to distinguish from the reads originating from low-frequency new transposon insertions (9).

Many algorithms have been developed to detect transposon insertions, including ERVcaller, MELT, RetroSeq, RelocaTE2 and TEMP, with TEMP being developed by us previously (10–15). All of these algorithms identify transposon insertions using the same strategy—clustering discordant read-pairs, which have one read mapping to the reference genome and the other read mapping to a transposon consensus sequence. These algorithms perform similarly well in detecting typical transposon insertions, and the differences between them lie in the ability to filter out false-positive predictions and to identify atypical insertions, those of low frequencies and those from short or homologous transposons. MELT, which was developed as part of the 1000 Genomes Project, carefully identifies discordant and split read-pairs for transposon detection. (Split read-pairs map to just one location of the genome but the 5′-end of one read is soft-clipped.) It is able to detect transposon insertions in low sequencing-depth data, but it also makes many false-positive predictions. ERVcaller and TEMP are stricter at assessing discordant and split read-pairs, which leads to higher precision. RetroSeq and TEMP are primarily designed for long transposons and are not suitable for detecting insertions of short transposons. Each of these tools works well on specific datasets or on specific transposons, but it is unclear whether they work well across a broad range of datasets and on all transposons; this is because there are few benchmark datasets for algorithm comparison. Moreover, the existing tools do not have the functionality to identify *de novo* transpositions, which occur in a single generation and reflect on-going transposon activity.

This paper makes two contributions. First, we present a new software TEMP2, which combines the best components of existing algorithms for detecting germline transposon insertions and applies thorough filtering steps to reduce false-discovery predictions while maintaining a high sensitivity. TEMP2 has a new functionality of estimating the rate of *de novo* transposon insertions, taking advantage of the knowledge that discordant read-pairs that truly originate from recent transposon insertions should be enriched in the ends of full-length transposons. Second, we performed PacBio long-read sequencing and Illumila short-read sequencing in *Drosophila melanogaster* and curated a set of transposon insertions using the PacBio data. These two datasets should be useful for benchmarking the performance of algorithms for detecting transposon insertions. Evaluated using the aforementioned state-of-the-art transposon algorithms on simulated data in flies, our PacBio-Illumina fly data, and human data from the 1000 Genomes Project, TEMP2 showed the best overall performance regarding sensitivity, precision, frequency accuracy, breakpoint accuracy, and transposon-end accuracy. We applied TEMP2 to estimate *de novo* insertion rates in simulated and hybrid dysgenic fly data, revealing the on-going transposon activities while these flies recover from the initial deficiency in piRNA production and transposon derepression. TEMP2 is freely available at Github: https://github.com/weng-lab/TEMP2.

## MATERIALS AND METHODS

### The TEMP2 pipeline

**Figure 1.**
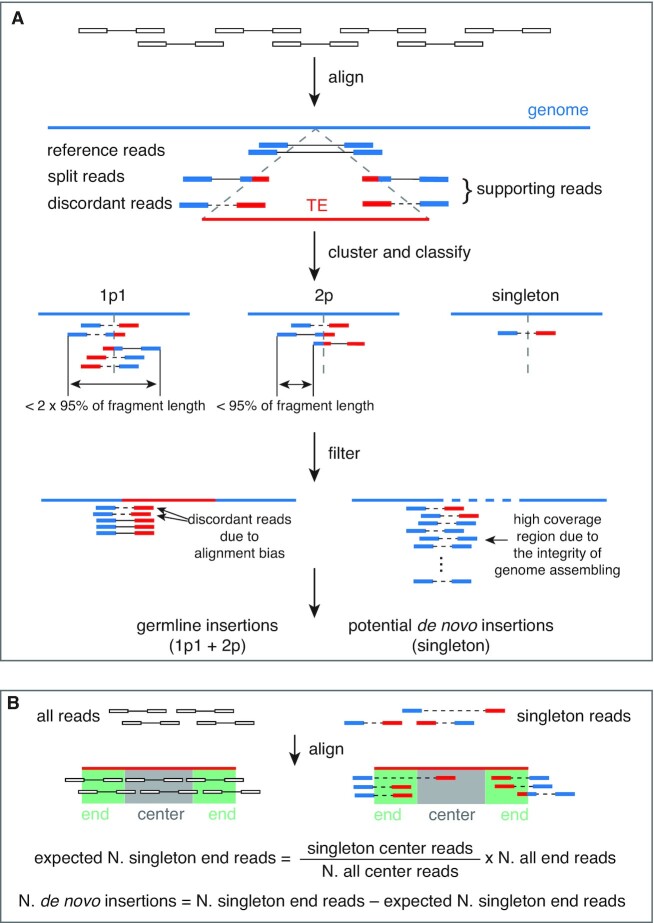
Diagrams depicting how TEMP2 detects new germline and *de novo* transposon insertions. (**A**) Detection of new transposon insertions. The method contains three steps: alignment, clustering/classifying, and filtering. Paired-end reads are depicted as pairs of boxes each connected by a short horizontal line: open boxes for unmapped reads and colored boxes for mapped reads. The reference genome is represented as a blue line while a transposon element (TE) as a red line. The portion of a read mapped to the reference genome is marked in blue and the portion of the read mapped to a transposon is marked in red. Properly mapped read-pairs are connected by solid lines while discordant read-pairs are connected by dashed lines. Transposon-supporting read-pairs that are anchored at the same genomic locus (defined as within 95% of the fragment length of the sequencing library) are clustered. A 1p1 cluster is supported by multiple read-pairs with at least one read-pair on each side of the transposon insertion, a 2p cluster is supported by two or more read-pairs, but only on one side of the insertion, and unclustered read-pairs are singletons. (**B**) Estimation of the total number of *de novo* insertions of a transposon family. All raw reads (empty boxes) and singleton reads as defined in A. (colored boxes) are aligned to the consensus sequence of each transposon family. According to where in the consensus the reads map, they are classified as end-mapping reads and center-mapping reads (see Materials and Methods). The total number of *de novo* insertions of a transposon family is defined as the difference between the actual number of end-mapping singleton reads and the expected number of end-mapping chimera reads, with the latter estimated using center-mapping singleton reads and all reads.

The overall pipeline of TEMP2 is shown in Figure 1, containing three steps described as follows.

The first step of TEMP2 is mapping reads to the reference genome using the bwa mem algorithm (16) with the following command: bwa mem -T 20 -Y. Two types of read-pairs are then extracted from the mapping results: (i) discordantly mapped read-pairs for which one read is uniquely mapped to the reference genome while the other read is unmappable or mapped to multiple locations in the genome (Supplementary Figure S1; read-pairs #1–#8, #13–#36, and #41–#56); (ii) split read-pairs that are properly mapped to just one location of the genome but the 5′-end of one read is soft-clipped (Supplementary Figure S1; read-pairs #9–#12 and #37–#40). The unmappable, multiply mapped reads, and split reads are then aligned to transposon consensus sequences using bwa mem, and the read-pairs that can be mapped to transposons are considered as reads that can support transposon insertions.

The second step of TEMP2 is clustering and classifying. Two transposon-supporting reads are placed into the same cluster if they satisfy either of the following two conditions: (i) they map to the same side of a transposon insertion and the distance between their mapped locations in the genome is smaller than the 95% quantile of the fragment length of the sequencing library, or (ii) they map to the opposite sides of a transposon insertion and their distance is smaller than twice the 95% quantile of the fragment length of the sequencing library. We then determine the breakpoints of an insertion using the soft-clipping site of split reads. If no split reads are available, we set the average coordinate of the 3′-ends of the supporting reads (Figure 1A) as the breakpoint. All insertions supported by read clusters are classified into three types according to their genomic location and read count: 1p1 (one-plus-one) insertions are supported by read-pairs on both sides of the insertion; 2p (two-plus) insertions are supported by two or more read-pairs but these reads all come from one side of the insertion; and *de novo* insertions are supported by only one (i.e. singleton) read-pair. TEMP2 considers 1p1 and 2p insertions as germline insertions that are passed on to the next generation and uses singleton read-pairs to estimate the level of *de novo* insertions, which include the insertions into somatic genomes or the insertions into the germline genomes that do not lead to offspring. TEMP2 also allows users to set an insertion-frequency threshold for classifying whether an insertion is *de novo*, which is necessary when the sequencing library is constructed from a small number of cells because in such cases *de novo* insertions may be supported by multiple reads due to PCR amplification of the small amount of starting DNA.

The third step of TEMP2 is filtering. Three types of filtering are applied to remove false-positive insertions. First, TEMP2 discards insertions by a transposon into a location in the genome that is annotated to contain a copy of the same transposons, because the discordant reads that support such insertions are likely due to sequence alignment errors. Furthermore, we place these insertion positions on a blacklist to filter out other insertions detected at the same genomic positions, which often come from transposons in the same family, again, suggesting alignment errors. Second, TEMP2 estimates the sequencing depth in the genomic region around each candidate insertion and compares it with the average sequencing depth across the whole genome. The number of mapped genome-sequencing reads that fall in a genomic window follows a bimodal distribution with one mode around the average coverage and the other mode much higher than five times the average coverage (Supplementary Figure S2A). Specifically, in our Illumina sequencing data, 0.226% genomic windows had 5× or more reads than the overall genome coverage (27.1×). Thus, we filtered out the insertions located in genomic regions with 5× or higher sequencing depths. Third, TEMP2 merges the insertions at exactly the same genomic position—the vast majority of these insertions are from related transposon subfamilies—and assigns all supporting reads to the insertion with the most supporting reads. We conducted these three filtering steps immediately after calling potential transposon insertions to minimize the number of insertions and insertion-containing genomic regions that we need to examine, reducing TEMP2’s runtime.

After identifying germline transposon insertions, TEMP2 also estimates the frequency for each transposon insertion. Properly mapped unsplit read-pairs crossing more than 20 bp of an insertion breakpoint are defined as reference read-pairs. The frequency of each transposon insertion is estimated using the equation below:

$$\begin{equation*}{\rm insertion}\ {\rm frequency} = \frac {{\rm supporting}\ {\rm reads}} {( {{\rm supporting}\ {\rm reads}\ + \ {\rm reference}\ {\rm reads}\ \times \ 2})}\end{equation*}$$

TEMP2 estimates the overall level of *de novo* transposon insertions for each transposon family in the whole genome using transposon-supporting singleton reads; however, TEMP2 does not make predictions on transposon insertions at individual loci. To detect *de novo* insertions, TEMP2 must guard against the chimera reads introduced during library construction, which are often singletons. Chimera reads should map to all locations in a transposon consensus sequence uniformly, while singleton reads that support transposon insertions should be enriched in the two ends of the transposon consensus sequence, as far into the interior of the consensus sequence as the fragment length of the sequencing library would allow. Thus, we can use singleton reads that map to the center region (the consensus sequence minus the two ends) to estimate the number of chimeric reads. TEMP2 determines the fragment lengths for all the read-pairs that map entirely to a unique location in the reference genome and then defines the end of a transposon as the 95th percentile fragment length minus 25 nts. The number of *de novo* insertions of a transposon family can be inferred by the difference between the number of end-mapping singleton reads and the number of center-mapping singleton reads; thus, the overall level of *de novo* insertions of a transposon family is:

$$\begin{equation*}{\rm singleton\ end\ reads} - {\rm singleton\ center\ reads}\, \left( \frac {{\rm all\ end\ reads}} {{\rm all\ center\ reads}}\right )\end{equation*}$$

TEMP2 outputs a confidence score (ranging from 0–100%) for each transposon family that equals one minus our estimated overall rate of chimera reads for this transposon family. Supplementary Figure S2B uses two example transposons to illustrate how TEMP2 estimates *de novo* insertion frequencies. Using our Illumina sequencing data, TEMP2 estimates that *roo* does not have a higher than the background level of *de novo* insertions because its total number of singleton end–reads does not exceed the expected number of singleton end–reads, while *Tirant* is estimated to have 43 *de novo* insertions.

In a typical application when a sufficiently large number of cells (thousands or more) is used in the starting material to prepare the sequencing library, TEMP2 only considers singleton insertions as potential *de novo* insertions to estimate the genome-wide *de novo* insertion rate. In the rare cases when a limited number of genomes (hundreds or less) is used in the starting material, TEMP2 will not just consider singleton insertions, but will instead ask the user to provide the number of genomes in the starting material and then automatically set the insertion frequency threshold to be two times the theoretical frequency of *de novo* insertions for distinguishing potential *de novo* insertions from germline insertions.

To account for the cases of truncated *de novo* insertions such as 5′ truncated L1 elements (15,17,18), TEMP2 can also classify singleton reads that map to the two ends of fragmented transposons as insertion-supporting reads (using the ‘-T’ option), if there are enough reads (by default three or more reads at each end) to support these fragmented transposons elsewhere in the genome. Such fragmented transposons are used together with full-length transposons in the same family for computing end-mapping reads and center-mapping reads in the above equation for computing the overall rate of *de novo* insertions.

### Simulated data

To benchmark the performance of TEMP2 and other transposon-detection methods, a set of Illumina sequencing data was simulated (see Supplementary Figure S3 for a summary). We simulated genomes with 400 new germline transposon insertions at different frequencies (0.25, 0.5, 0.75 and 1) and insertion lengths as follows. We first constructed 10 000 reference genomes (dm6) and then inserted 90 full-length transposons (randomly picked) and 10 partial-length transposons (6 *I-element*, 2 *Doc*, 2 *F-element*) into the same coordinates of 2500, 5000 or 7500 of the 10 000 reference genomes one by one. We also simulated 10 000 genomes with 20 somatic transposon insertions each. We inserted eight full-length *297*, four full-length *copia*, three full-length *Tirant*, two partial-length *Doc*, one full-length *17.6*, one full-length *F-element* and one full-length *blood*, hence 20 transposons in total, into different coordinates of the 10 000 simulated genomes one by one. Low mappability regions were excluded when inserting transposons.

Illumina read-pairs were then simulated using the ART algorithm (version 2.5.1) with parameters -ss HS25 -p -l 100 (read length) -m 450 (fragment size) -s 10 -na (19). For each of the 10 000 simulated genomes, we simulated Illumina read-pairs at 0.0001×, 0.0002×, 0.0003×, 0.0004×, 0.0005×, 0.001×, 0.002×, 0.003×, 0.004× and 0.005× genome coverage by adding parameter -f. In total, Illumina read-pairs at the sequencing depth of 1–50× genome coverage for 10 000 simulated genomes were generated for each genome set. Not that by 1–50× genome coverage, we mean that the total number of nucleotides that mapped to the reference genome was at 1–50× the genome length. Two additional datasets with different percentages of chimera read-pairs (0.05% and 0.5%) were generated by combining two random reads into one read-pair.

### PacBio and Illumina whole-genome sequencing of *Drosophila*

For PacBio sequencing, the female virgin flies (ISO-1 strain, ∼180 individuals for each of two samples) were collected and starved for 1.5 h and flash-frozen in liquid nitrogen. Genomic DNA was extracted and purified with standard procedures. The DNA library preparation for PacBio sequencing was performed by following the PacBio protocol called ‘procedure & checklist of 20 kb template preparation using the BluePippin size-selection system’. Briefly, the DNA was sheared by a Covaris g-TUBE device and purified using AMPure PB beads. The fragmented DNA was subject to DNA damage repairing and ligated with adapters. Then purified ligation products were size-selected using the BluePippin Size Selection system. After annealing and binding of SMRTbell templates and preparation for MagBead loading, the two libraries were run on the PacBio RS II and Sequel system in NextOmics (Wuhan, China), respectively. The sequencing results for each sample contained two SMART cells.

For Illumina short-read sequencing, the whole bodies of 3–5-day-old female virgin flies (ISO-1 strain, ∼25 individuals) were collected and used for DNA extraction. DNA quality was assessed by OD260/OD280 with Nanodrop and agarose gel electrophoresis. The library for Illumina sequencing was prepared as follows: (i) fragmentation with Covaris ultrasonicator, (ii) end-repair and phosphorylation of the 5′ ends, (iii) A-tailing of the 3′ ends, (iv) ligation of adapters, (v) 12 cycles of PCR to enrich for the ligated product. Sequencing was done with the Illumina HiSeq-2500 sequencer (run type: paired-end; read length: 125 nt) in Novogene (Tianjin, China).

### Build a benchmark of transposon insertions using PacBio sequencing data

PacBio sequencing data were transformed to the FASTA format and then aligned to the dm6 genome using the Minimap2 algorithm (version 2.16) with parameters -x map-pb –MD (20). The mapping result was then provided to the Sniffles algorithm for structural variation detection with parameters -l 300 -s 1 (21). Only insertions longer than 300-bp were retained for further analysis because the shortest transposon in *D. melanogaster* is *Stalker3*, which is 372-nt in full length. The sequences of insertions were extracted and aligned to transposon consensus sequences using Minimap2 again to define new transposon insertions. A new transposon insertion is considered valid if both of the following conditions are satisfied: (i) the aligned length is longer than half of the insertion (ii) the alignment starts within 500-nt of the 5′-end of the insertion and ends within 500-nt of the 3′-end of the insertion. Transposon insertions within 50 bp were merged, and insertions with more than one supporting read were retained and considered as germline transposon insertions. Breakpoints of the insertions were set to the insertion sites that were supported by the most reads. The 5′-end and 3′-end of each inserted transposon were also annotated. To estimate insertion frequencies, genome-mapping PacBio reads around each breakpoint were tallied. Reads that cross a breakpoint for at least 50 bps were defined as reference reads, and reads split within 50 bp of the breakpoint were defined as supporting reads. Some PacBio reads were long enough to split in both the 5′-end and the 3′-end of an insertion, and these reads were counted as two supporting reads. The insertion frequencies were then estimated using the same equation as TEMP2:

$$\begin{equation*}{\rm{insertion\ frequency\ }} = \frac {{\rm{\ supporting\ reads\ }}} {\left( {{\rm{supporting\ reads\ }} + {\rm{\ reference\ reads\ }} \times {\rm{\ }}2} \right)}\end{equation*}$$

We then manually inspected each of the 405 transposon insertions detected using the PacBio data. Among these 405 insertions, 73 were located in an annotated copy of the same transposon in the reference genome. We visualized the PacBio raw reads supporting each insertion using the IGV browser (v2.7.2) to examine detailed alignments (22). Furthermore, we manually aligned each inserted sequence back to the transposon consensus sequence. For 11 high-frequency insertions supported by many PacBio reads, the insertion sites made by the supporting PacBio reads were typically at exactly the same location of the reference genome or within a few base-pairs of each other, indicating that these are true insertions. For the remaining 62 insertions, a portion of the supporting PacBio read could not align to the reference genome due to high sequencing errors in the portion. However, when we manually aligned the portion back to the transposon consensus sequence, more than half of the portion could be aligned. Furthermore, their supporting PacBio reads point to positions in the reference genome that were far from one another (hundreds to thousands of base pairs away), suggesting alignment errors. We deemed these insertions false positives. We further examined whether the 332 PacBio-detected insertions that were not in a copy of the same transposon could be supported by any Illumina reads. We first aligned Illumina reads to the reference genome via bwa mem using default parameters and then identified discordantly mapped read-pairs from the ± 500 bp region flanking each of the 332 insertions. We aligned these discordant read-pairs to transposon consensus sequences via bwa mem using default parameters. If there was at least one discordant read-pair that could align to the inserted transposon, we deemed the insertion supported by Illumina reads.

### Algorithm comparison

The main differences between the algorithms assessed by us are listed in Supplementary Table S4. Algorithms were benchmarked on three sets of short-read whole-genome sequencing data: simulated *D. melanogaster* data, experimental *D. melanogaster* data we produced, and human data in the NA12878 lymphoblastoid cell line downloaded from the 1000 Genomes Project.

For simulated and *D. melanogaster* data, default parameters for each algorithm were used. To achieve a fair comparison of the algorithms, the same cutoff of transposon-supporting reads were used for each of the algorithms (five reads). Sum of squared residue (SSR) was defined as the sum of errors of estimated *de novo* insertion rate across all transposons including the transposons with 0 simulated insertions:

$$\begin{eqnarray*}\mathop \sum \limits_{i\ = \ 1}^n {({\rm estimated\ }de\ novo{\ \rm insertion\ number\ per\ genome}}\nonumber\\ - {\rm{simulated\ }}de\ novo{\rm{\ insertion\ number\ per\ genome})^2}\end{eqnarray*}$$

**Figure 2.**
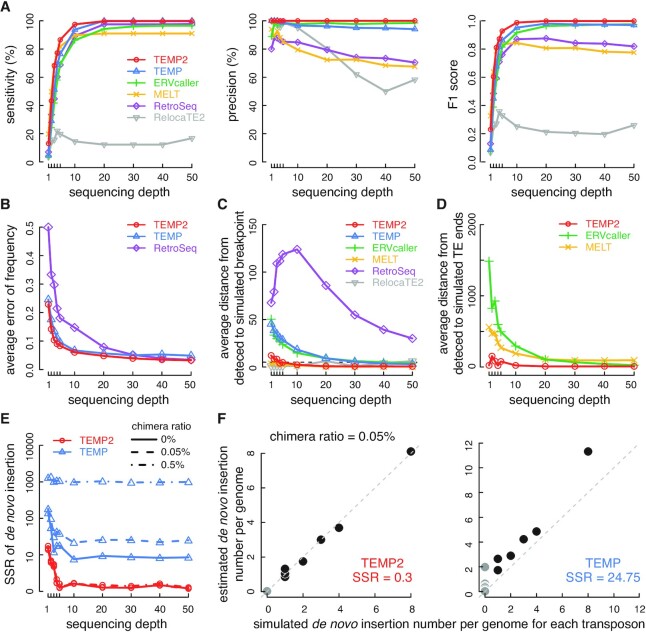
The performance of TEMP2 on simulated datasets. Simulated Illumina read-pairs at different sequencing depth (1–50× genome coverage) were used for comparing the performance of TEMP2, TEMP, ERVcaller, MELT, RetroSeq and RelocaTE2 (in red, blue, green, yellow, purple and gray respectively). Panels A–D, germline insertions. Panels E-F, somatic insertions. Except for panel E, for which three levels of chimera read-pairs were tested, the datasets with 0.05% chimera read-pairs were used for all other panels. (**A**) Performance of TEMP2 and other transposon-detection methods in detecting transposon insertions. Three panels of line plots depict the sensitivity, precision, and F1 score of detecting germline transposon insertions, respectively, as a function of sequencing depth. (**B**) Accuracies of TEMP2, TEMP and RetroSeq in estimating transposon-insertion frequencies. Line plots show the average error of estimated frequencies of germline transposon insertions as a function of sequencing depth. (**C**) Accuracies of TEMP2 and other transposon-detection methods in identifying the breakpoints in the reference genome. Line plots show the average distance between detected and simulated breakpoints of new germline transposon insertions. (**D**) Accuracies of TEMP2 and two other transposon-insertion methods in predicting the ends of inserted transposons. Line plots show the average distance between detected and simulated transposon ends of new germline insertions. (**E**) Accuracies of TEMP2 and TEMP in estimating somatic transposon insertion numbers. Line plots show the sum of squared residuals (SSR) of estimated somatic insertion numbers for all transposon subfamilies. Simulated data with 0%, 0.05%, and 0.5% chimera were tested and the results are displayed as solid, dashed and dot-dashed lines respectively. This panel and panel F are benchmarked using simulated *de novo* insertions from six full-length transposons and one fragmented transposon (*Doc*). (**F**) Accuracies of TEMP2 and TEMP in estimating somatic transposon insertion numbers; the sequencing depth was set to 20×. Scatterplots compare simulated and estimated insertion numbers. Each dot denotes a transposon subfamily, and the 8 transposon subfamilies with simulated somatic insertions are in black while the other transposon subfamilies are in gray.

The SSRs were 0.3 for TEMP2 and 24.75 for TEMP (Figure 2F). When we considered only those seven transposons with non-zero simulated insertions, SSRs were 0.3 for TEMP2 and 17.69 for TEMP. The transposon library of *D. melanogaster* was downloaded from Flybase (23). Transposon insertions in the reference genome (dm6) were annotated using RepeatMasker with parameters -s -no_is -norna -nolow -e ncbi -cutoff 255 -div 40 -frag 20000 (24).

We downloaded the .cram or .bam file of NA12878 low-depth and high-depth data from the 1000 Genomes Project. Although TEMP2 can directly work with these files, we wanted to ensure that the same parameters were used for genome mapping, so we extracted raw reads from these files using samtools (25) and then aligned the reads to hg38 using bwa mem with parameters ‘-T 20 -Y’ (16). Default parameters for ERVcaller, MELT, RetroSeq were used to analyze the NA12878 data. We allowed 10% sequence divergence for TEMP2 and TEMP when aligning reads to transposon consensus sequences, the same for MELT. To achieve a fair comparison of the algorithms, the same cutoff of transposon-supporting reads were used for each of the algorithms (3 for low-depth data and 10 for high-depth data). The transposon library, which contains Alu, SVA, and LINE1 consensus sequences, was downloaded from the MELT package (10). The reference insertion annotation of Alu, SVA, and LINE1 was also downloaded from the MELT package.

## RESULTS

### Design and Implementation of TEMP2

TEMP2 is a complete redesign of our previous algorithm TEMP (26), which identifies transposon insertions using paired-end short reads. TEMP uses three steps to detect transposon insertions: alignment, clustering-classifying, and filtering. In TEMP2, we improved each of the steps to achieve higher accuracy in identifying germline transposon insertions and added a new module in TEMP2 for estimating the level of *de novo* transposon insertions (see Materials and Methods).

TEMP2 has improved upon TEMP in all three steps (Figure 1A). In the first alignment step, discordant read-pairs, of which one read uniquely maps to the genome and the other maps entirely (unsplit) or partially (split) to distant transposon copies, are identified as possibly supporting a new transposon insertion in the genome (Supplementary Figure S1). TEMP only considers the unsplit reads, while TEMP2 additionally includes the split reads if their non-transposon mapping portions properly map to the genome, i.e. in the same orientation and within a distance cutoff defined as the 95th percentile of the fragment length of the sequencing library (see Materials and Methods). In the second clustering-classifying step, supporting reads are clustered, and the insertions supported by the read clusters are classified by genomic location and read count. Like TEMP, TEMP2 classifies insertions into three types: 1p1, supported by read-pairs on both sides of the insertion; 2p, supported by two or more read-pairs but on only one side of the insertion; and singletons, supported by only one read-pair. Going beyond TEMP’s classification, TEMP2 further deems 1p1 and 2p insertions as germline insertions but examines singleton read-pairs for estimating the genome-wide level of *de novo* transposition for each transposon family (a new module described next). In the third filtering step, some insertions are filtered out if they are likely false positives. Like TEMP, TEMP2 discards insertions of a transposon into an annotated copy of the same transposon in the genome because the supporting discordant reads are likely misaligned to this annotated copy due to the variations among the copies of the same transposon. TEMP2 further filters out insertions in regions that have 5× or higher sequencing depth (Materials and Methods). Such regions are determined for each specific dataset and likely reflect the incompleteness of the reference genome with respect to the genome that corresponds to the sample being assayed. Moreover, we observed that some genomic positions contained insertions from multiple transposons, and the vast majority of these insertions were from related transposon subfamilies. We observed this phenomenon even in simulated data where no TE subfamilies were simulated to be inserted in the same locus and at roughly the same frequency in our Illumina sequencing data as in the simulated data. These insertions are caused by ambiguous mapping of reads to related transposon subfamilies. Thus, TEMP2 merges the insertions at exactly the same genomic position and assigns all supporting reads to the transposon with the most supporting reads.

We developed a new module in TEMP2 to estimate the overall level of *de novo* insertions for each transposon family. Such *de novo* insertions can be in somatic cells or in the germ cells that do not develop into the next generation of individuals. The identification of *de novo* insertions is challenging; they are often present in only one cell or a few cells derived from a single progenitor, and therefore present at very low frequencies and dispersed throughout the genome, and thus represented by single read-pairs in the library. As a result, it is difficult to distinguish discordant reads supporting real *de novo* insertions from chimeric reads introduced during sequencing library construction (9). However, new transposon insertions have terminal repeats that are in a fixed orientation relative to the junction of inserted transposons with genomic sequence, and short supporting read-pairs should only align to the ends of transposons, and have the correct orientation (Figure 1B). We, therefore, used read-pairs mapping to the two ends of a transposon (defined as the 95th percentile of fragment length of the library minus 25 nucleotides or nts, see Materials and Methods) to estimate *de novo* insertions, and assume that read-pairs that map to the center of the transposon are chimeric reads. Thus, TEMP2 defines the total number of *de novo* insertions of a transposon as the difference between the number of end-mapping singleton reads and the expected level of chimeric reads, which is estimated from experimentally defined reads mapping to the center of the same transposon family (Figure 1B).

### Performance assessment using simulated datasets

We generated a series of simulated datasets to compare the performance of TEMP2 and several other transposon-detection algorithms. We first simulated 10 000 *Drosophila melanogaster* genomes by inserting germline and somatic transposons (Supplementary Figure S3; Supplementary Table S1; see Materials and Methods), with each germline insertion at the same position across all the genomes while somatic insertions at different locations among the genomes. To evaluate the accuracy of insertion frequency estimations by the algorithms, germline insertions were simulated with frequencies from 0.25 to 1, i.e. 25% to 100% of the 10 000 genomes would contain an insertion. We also generated 10% of the insertions as transposon fragments to evaluate the accuracy in predicting full-length insertions (see Materials and Methods). Finally, we simulated Illumina read-pairs at different sequencing depths (1–50× genome coverage) with different levels of chimera read-pairs (0%, 0.05% and 0.5%) from the 10 000 simulated *Drosophila* genomes with the short-read simulation tool ART (19).

We evaluated the performance of TEMP2, TEMP and four other transposon-detection algorithms: ERVcaller, MELT, RetroSeq and RelocaTE2 (10,26–29) with five metrics for detecting germline transposon insertions: sensitivity and precision in detecting insertions, accuracy in estimating insertion frequencies, accuracy in identifying the breakpoints in the reference genome, and accuracy in predicting the two ends of inserted transposons. All six algorithms report the number of supporting reads for each detected transposon insertion, but they apply different filtering to decide on the final predicted insertions. To achieve a fair comparison of the algorithms, we performed the assessment using the same cutoff of supporting reads for all the algorithms. We evaluated the performance of the algorithms as a function of the total sequencing depth because all algorithms performed better at higher sequencing depths. Figure 2 shows the results at five or more supporting reads and 0.05% chimera read-pairs; we also tested the cutoff of three or four supporting reads and 0% or 0.5% chimera read-pairs and reached the same conclusion (Supplementary Table S2).

TEMP2, TEMP and ERVcaller achieved higher sensitivity and precision than the other three algorithms in detecting germline transposon insertions across 1–50× sequencing depths (Figure 2A). At the typical 20× sequencing depth, TEMP2 (sensitivity 99.8% and precision 100%) and TEMP (100% and 96.2%) showed the highest sensitivity and precision, followed by ERVcaller (94.3% and 98.4%), RetroSeq (97.5% and 74.9%), MELT (91% and 72.4%), and RelocaTE2 (12.3% and 80.3%). TEMP2 maintains perfect precision throughout the sequencing depths. ERVcaller applies several filtering steps, which may explain its steadily high precision (27).

We compared the accuracy in estimating insertion frequencies for the three algorithms that provide such an estimation: TEMP2 and TEMP output insertion frequencies directly, and RetroSeq outputs the number of transposon-supporting reads and reference reads which can be used to estimate insertion frequencies (see Materials and Methods). The error in estimating insertion frequencies went down with increasing sequencing depth for all three methods (Figure 2B; Supplementary Figure S4A), and TEMP2 shows the lowest error (average error = 0.048 at 20× sequencing depth).

The breakpoints in the reference genome correspond to the locations where transposon insertions occurred. We quantified the accuracy in breakpoint identification using the average distance between simulated breakpoints and the breakpoints identified by each algorithm (Figure 2C). TEMP2, MELT and RelocaTE2 identified accurate breakpoints. The average distance was less than ten base-pairs (bp) for TEMP2 at sequencing depths higher than 3×.

New transposon insertions are expected to be full length, but they degenerate over time and most copies in the genome are fragments. We measured the accuracy in predicting the ends of inserted transposons by calculating the average distance between detected and simulated transposon ends (Figure 2D). Only TEMP2, MELT and ERVcaller predict the ends of each inserted transposon. TEMP2 achieves small distances across the sequencing depths tested, while MELT and ERVcaller performed well only at high sequencing depths (>20×).

We then assessed the performance of TEMP2 in estimating the level of *de novo* transposon insertions. Although the singleton reads reported by TEMP can be used to estimate de novo insertions, other algorithms do not provide this functionality, possibly because the other algorithms treat singleton reads as unreliable information that is difficult to distinguish from chimera artifacts. We, therefore, compared TEMP2 with TEMP. We calculated the sum of squared residuals (SSR) between the detected and simulated somatic insertion numbers per genome, with the simulation performed at a series of sequencing depths and three different percentages of chimera read-pairs. TEMP2 achieved substantially lower SSR than TEMP, 0.3 versus 24.75 at 20× sequencing depth with 0.05% chimeric reads; furthermore, TEMP2’s SSR remained low at 0.5% chimera reads, while TEMP’s SSR was substantially higher at a higher chimera rate. We also performed the comparison for each transposon subfamily, and the somatic insertion numbers predicted by TEMP2 were nearly perfectly correlated with the simulated insertion numbers (Figure 2F and Supplementary Figure S4B, left panel, *r* = 0.998; *P*-value < 2.2 × 10^−16^) while TEMP estimated significantly higher levels of *de novo* insertions than the simulated levels due to chimeric reads (Figure 2F and Supplementary Figure S4B, right panel; Wilcoxon signed-rank test *P*-value < 2.2 × 10^−16^).

Finally, we compared the runtime of the six algorithms using eight threads and 32GB memory (Supplementary Figure S4C). As expected, the run time was positively correlated with the sequencing depth of the dataset. For determining germline insertions, TEMP2 runs the fastest among the six algorithms we benchmarked, followed by TEMP, Retroseq, ERVcaller, MELT, and RelocaTE2. For estimating *de novo* insertion numbers, TEMP2 spent 15.6 minutes on the dataset with 20× coverage of the *Drosophila* genome and 0.05% chimera read-pairs, comparable to the other algorithms which only detect germline insertions.

In summary, in comparison with five other algorithms, TEMP2 shows the best performance in sensitivity and precision of detecting germline transposon insertions, further showing the lowest errors in estimating insertion frequencies, identifying the breakpoints in the host genome, and predicting the ends of inserted transposons. Additionally, TEMP2 can accurately estimate the total number of *de novo* transposon insertions, but the other algorithms lack this functionality. TEMP2 is the fastest among the six algorithms we examined.

### A curated benchmark dataset of transposon insertions using PacBio and Illumina sequencing in *Drosophila melanogaster*

**Figure 3.**
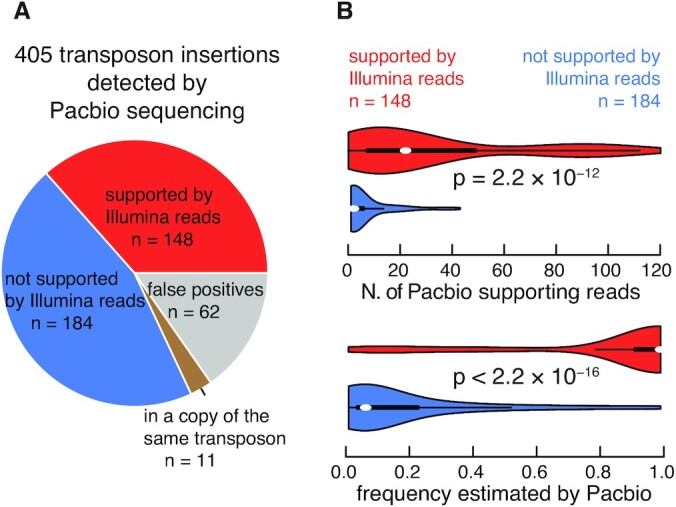
Constructing a benchmark set of transposon insertions using PacBio and Illumina sequencing data. (**A**) 405 transposon insertions detected by PacBio sequencing. A pie chart depicts the classification of 405 PacBio-detected transposon insertions into 148 insertions supported by Illumina reads (red), 62 false-positive insertions into a copy of the same transposon (gray), 11 manually-verified insertions into a copy of the same transposon (brown), and 184 insertions not supported by Illumina reads (blue). (**B**) Insertions supported by Illumina reads have more PacBio supporting reads and higher frequencies. Violin plots compare the number of PacBio-supporting reads (top) and insertion frequencies estimated by PacBio reads (bottom) for 148 insertions supported by Illumina reads (red) and 184 insertions not supported by Illumina reads (blue) as defined in A.

To benchmark the performance of transposon detection algorithms, we performed high-depth PacBio long-read (171× genome coverage) and Illumina short-read sequencing of *Drosophila melanogaster* (see Materials and Methods). In both Illumina and PacBio sequencing, we used the ISO-1 strain of *D. melanogaster*, which was inbred and sequenced to assemble the reference fly genome, to exclude the potential effect of heterozygous transposon insertions in transposon detection (30,31). The two sequencing libraries were built separately, using ∼25 female virgins for Illumina sequencing and ∼180 female virgins for PacBio sequencing. The PacBio sequencing reads were long enough to span entire transposons; therefore, they can be used to construct a benchmark of transposon insertions (see Materials and Methods). In total, we detected 405 transposon insertions that were not in the reference fly genome, along with their insertion frequencies quantified using the PacBio data (Supplementary Table S3), and these insertions were then subject to further analysis and manual curation to arrive at a gold-standard set of insertions (Figure 3A).

Among these 405 insertions, 73 insertions were from a transposon into an annotated copy of the same transposon in the reference genome. Among these, 54 insertions were supported by one PacBio read each, and manual curation revealed that these insertions were due to sequencing errors in the PacBio reads because the inserted fragment could map back to the same transposon, albeit with mismatches. The other 19 insertions were supported by multiple PacBio reads; however, manual inspection revealed that eight of them were false positives as well for a similar reason. The remaining 11 insertions appeared to be real (Figure 3A).

We examined the remaining 332 insertions for support by Illumina reads (see Materials and Methods), and 148 insertions had support while the remaining 184 insertions did not (Figure 3A). The 184 insertions not supported by Illumina reads had substantially fewer supporting PacBio reads then the 148 insertions supported by Illumina reads (Figure 3B, top panel)—114 of these 184 insertions were supported by only one PacBio read each, suggesting that they occurred only in one genome used to prepare the PacBio library. Furthermore, these two sets of insertions differ significantly in their insertion frequencies estimated by PacBio reads (Figure 3B, bottom panel; *P*-value < 2.2 × 10^−16^). Thus, the 184 insertions not supported by Illumina reads were likely insertions in a small subset of the genomes and, as a result, not replicated in the sample used to prepare the Illumina sequencing library. Thus, as a benchmark for testing algorithms that would use the Illumina reads as the input, we only included the 148 insertions that were supported by Illumina reads. We did not include in the benchmark the aforementioned 11 insertions into an annotated copy of the same transposon, because we could not use Illumina reads to verify them—full-length copies of the transposon elsewhere in the genome would produce Illumina reads to map to these locations. Furthermore, none of the algorithms tested could identify these 11 insertions.

More than half (*n* = 83) of the 148 Illumina-supported insertions contained more than 80% of the full-length consensus sequences of their transposons (Supplementary Table S3), suggesting that these insertions resulted from recent transpositions. Among the remaining 65 insertions, 27 were shorter than 20% of their consensus sequences; these are fragments of ancient insertions that have since degenerated.

### Detection of germline insertions in *Drosophila melanogaster*

**Figure 4.**
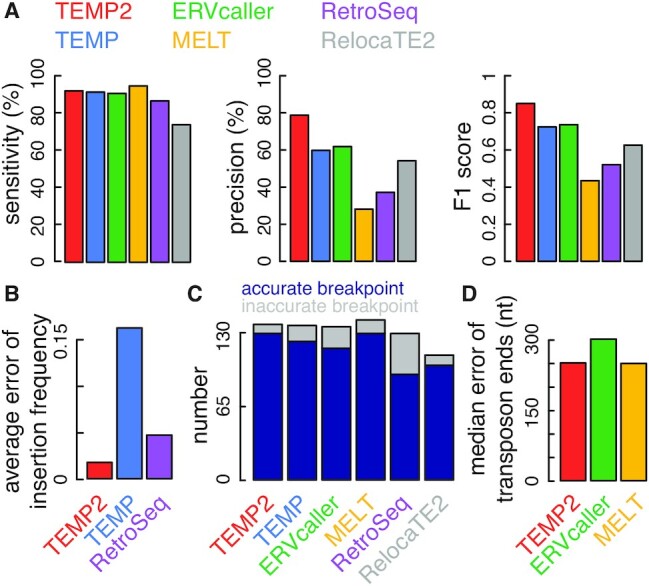
The performance of TEMP2 on experimental data in *D. melanogaster*. Genomic sequencing data from *D. melanogaster* at 29× sequencing depth were used for comparing the performance of TEMP2, TEMP, ERVcaller, MELT, RetroSeq and RelocaTE2 (in red, blue, green, yellow, purple, and gray respectively). (**A**) Performance of detecting transposon insertions. Three panels of bar plots depict the sensitivity, precision and F1 score of detecting germline transposon insertions, respectively. (**B**) Average errors of estimating transposon-insertion frequencies. Bar plots show the average error of estimated frequencies of germline transposon insertions. (**C**) Accuracies of identifying breakpoints in the reference genome. Bar plots show the number of germline transposon insertions (breakpoints) called by TEMP2 and other transposon-detection methods, with accurately detected breakpoints in dark blue and inaccurately detected ones in gray. (**D**) Accuracies in predicting the ends of inserted transposons. Bar plots show the median distance between detected and actual transposon ends of new germline insertions.

We used the 148 PacBio-detected, Illumina-supported transposon insertions as a benchmark to test TEMP2 and the other algorithms, which were provided the Illumina short-read sequencing data at 29× genome coverage. The four algorithms that achieved the highest sensitivities—MELT, TEMP2, TEMP and ERVcaller (95.3–91.2%; Figure 4A)—differed in their precisions: TEMP2 achieved 79.2% precision, MELT had low precision (28.3%), while TEMP and ERVcaller were in between (60.2% and 62.2%), and accordingly, TEMP2 achieved the highest F1 score of 0.854 (Figure 4A). We further compared the algorithms using three other metrics. Among the three algorithms that report insertion frequency, TEMP2 achieved the lowest average error of 0.072 (Figure 4B), judged by the insertion frequencies determined using the PacBio data (Supplementary Table S3). TEMP2 also detected the breakpoints accurately for the largest number of insertions (*n* = 129, 94.2% of the 137 insertions it predicted; Figure 4C). Among the three algorithms that predict the ends of inserted transposons, TEMP2 and MELT achieved lower errors than ERVcaller (Figure 4D). In summary, TEMP2 outperformed the other five algorithms overall judged by the 148 transposon insertions.

### Detection of germline insertions in human NA12878 cells

We further assessed the accuracy of TEMP2 and the other algorithms in detecting germline insertions in human lymphoblastoid cells, using two Illumina-sequencing datasets from the NA12878 donor in the 1000 Genome Project—one with low sequencing depth (5.7× genome coverage; 101-nt paired-end reads) and the other one with high sequencing depth (60× genome coverage; 250-nt paired-end reads). Note that these two datasets resulted from two distinct sequencing libraries that differed in fragment length (382 ± 36 nt for the low-depth dataset and 430 ± 154 nt for the high-depth dataset). We used the 893 validated germline transposon insertions as the gold standard, downloaded from a previous benchmark study for comparing transposon-detection algorithms (32). Most of these insertions (833 of 893; 93.3%) were Alu elements, and a full-length Alu element is 280-nucleotide long (33,34).

**Figure 5.**
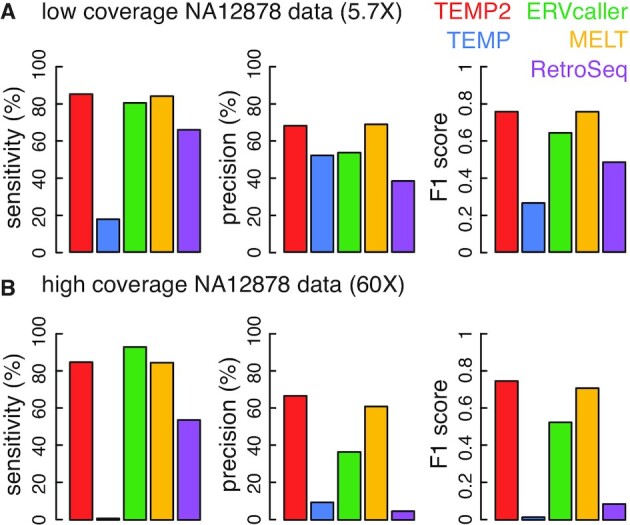
The performance of TEMP2 on NA12878 data from the 1000 Genomes Project. Genomic sequencing data sets of the NA12878 cell line at low (5.7×; panel **A**) and high (38.6×; panel **B**) sequencing depths were used for comparing the performance of TEMP2, TEMP, ERVcaller, MELT, and RetroSeq (in red, blue, green, yellow, and purple, respectively), in detecting germline transposon insertions. In each sub-figure, Three panels of bar plots depict sensitivity, precision and F1 score of detecting germline transposon insertions, respectively.

TEMP2 and MELT performed equally well for the low-depth dataset (F1 score = 0.758 and 0.757; Figure 5A). MELT was developed as a part of the 1000 Genomes Project for annotating transposon insertions, and it was trained on these datasets, consistent with its good performance. TEMP2 identified 761 of the 893 (85.2%) transposon insertions from the low-depth data at a 68.2% precision (Figure 5A). MELT achieved a slightly lower sensitivity of 84.1% and a slightly higher precision of 68.9%. ERVcaller was worse (80.4% sensitivity and 53.6% precision), followed by RetroSeq and TEMP (Figure 5A). TEMP was originally developed for detecting transposons in *Drosophila*, which are mostly longer than 1,000 nucleotides at full length; as a result, TEMP missed most of the short Alu elements in the NA12878 dataset. RelocaTE2 was omitted from the comparison because it did not finish after running for a week.

TEMP2 was slightly worse for the high-depth dataset (F1 score = 0.750) than for the low-depth dataset, with the decrease coming from the precision (Figure 5B). MELT showed a bigger decrease in performance (F1 = 0.711) than TEMP2, also with precision taking the hit (Figure 5B). ERVcaller achieved a higher sensitivity for the high-depth dataset than for the low-depth dataset (93.3% versus 80.4%), but a greater decrease in precision (53.6–36.6%; Figure 5B). RetroSeq and TEMP showed worse sensitivity and precision on the high-depth dataset. Since the high-depth dataset had a longer read length (pairs of 250-nt reads) than the low-depth dataset (pairs of 101-nt reads) and most insertions were Alu elements, which are only 280-nt long at full length, some of the Alu insertions were entirely contained in the read-pairs. To investigate the impact of sequencing depth and read length, we downsampled the high-depth dataset to mimic the short-depth dataset—87 million pairs of randomly sampled read with each read clipped at its 3′-end to 101-nt long, resulting in 5.7× genome coverage (Supplementary Figure S5A). Now RetroSeq performed similarly to what it did for the low-depth dataset, although TEMP mainly improved its precision. Without clipping the reads, which corresponded to a 14.1× genome coverage, RetroSeq and TEMP did worse than when the reads were clipped (Supplementary Figure S5B), confirming that these two methods have difficulty in finding the insertions of short transposons when the reads are long.

In summary, TEMP2 and MELT showed the best performance on identifying short-transposon insertions in human lymphoblastoid cells, and they performed reliably well on two datasets with different sequencing depths, read lengths, and fragment lengths.

### Detection of *de novo* transposon insertions in hybrid-dysgenic flies

To assess TEMP2’s newly developed ability to estimate the total number of *de novo* transposon insertions, we applied it to our previously published Illumina-sequencing dataset on the dissected ovaries of *Drosophila melanogaster* dysgenic hybrids (35). The sequenced samples included a wild-type strain *Harwich* (*Har*), a lab strain *white 1* (*w^1^*), first-generation progeny (F1) from crossing *Har* males with *w^1^* females (*wH*, two samples were prepared, from 2–4-day females and 21-day females, respectively), and second-generation progeny (F2) from crossing *w^1^* males with F1 females (*wHw*, one sample was prepared, from 21-day females). *Har* possesses *P-elements* in its germline genome, which are not present in the lab strain *w^1^*. F1 flies lack maternal piRNAs targeting paternal *P-elements*, which results in *P-element* activation, genome instability, and sterility. Surviving F1 females recover partial fertility as they age, producing *P-element*-targeting piRNAs *de novo*, and F2 females are fertile (35). Thus, TEMP2 can be used to assess the number of transposon insertions in these fly ovaries that were not passed on to the next generation.

**Figure 6.**
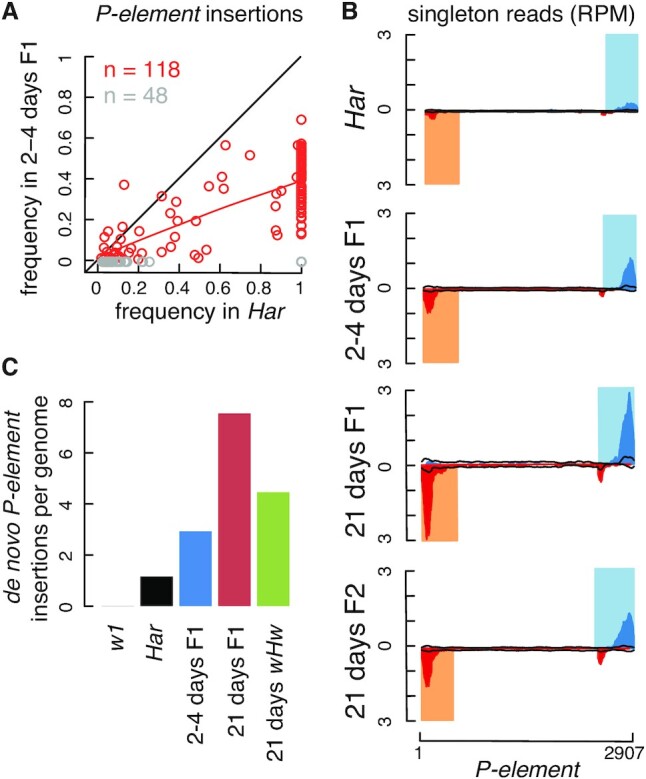
The performance of TEMP2 on the *D. melanogaster* P-M dysgenic datasets. (**A**) Germline frequencies of *P-elements* in F1 flies follow Mendelian genetics. These 2–4-day-old F1 flies were born to *Harwich* (*Har*) fathers who harbored germline *P-elements* and *w^1^* mothers who did not. The scatter plot compares the frequencies of germline *P-element* insertions in the *Har* flies vs. those in the F1 flies. Each dot denotes a *P-element* insertion supported by at least five reads in *Har* flies. The *P-element* insertions that were detected in F1 flies are in red while the undetected insertions are in gray. An *x* = *y* diagonal line is included as a reference, and a red line indicates the Loess regression of the red points, whose slope is about 0.5. (**B**) *P-elements* are actively transposing in F1 flies. Profiles show the numbers of singleton read-pairs that support *P-element* insertions in *Har*, 2–4-day F1, 21-day F1 and 21-day F2 (*wHw*) fli*es*. The portions of reads in insertion-supporting read-pairs that mapped to the sense (antisense) strand of the P-element consensus sequence are tallied to make the blue (red) curve (see Materials and Methods for details). The two ends of *P-element* are highlighted in light blue and light orange. Two black lines indicate all raw reads mapped to the *P-element* consensus sequence (sense and antisense, respectively), which were used to perform normalization while estimating *de novo P-element* insertions. (**C**) Estimated numbers of *de novo P-element* insertions per genome in P–M dysgenic datasets. Bar plots show the numbers of *de novo P-element* insertions per genome in *w^1^*, *Har*, 2–4-day F1, 21-day F1 flies and 21-day F2 (*wHw*) flies.

TEMP2 identified 166 *P-element* germline insertions with at least five supporting reads in *Har* ovaries, and 118 of them were also detected in 2–4-day F1 flies, typically with halved frequencies, in accordance with Mendelian genetics (Figure 6A). We then estimated the *P-element* insertion numbers in *w^1^*, *Har*, 2–4-days F1, 21-days F1 and 21-days F2 files that were not passed on to the next generation. Although *Har* flies possessed the largest number of *P-elements* in their germline genome, they showed very few *P-element*-mapping singleton reads, consistent with their possession of *P-element*-targeting piRNAs. In contrast, F1 flies had large numbers of *P-element*-mapping singleton reads, more in 21-day flies than in 2–4-day flies (Figure 6B), suggesting that *P-elements* were actively transposing in these flies without the repression by the piRNAs that target them. Note that the numbers of singleton reads that map to the interior region of the *P-element* consensus sequence in these four samples were roughly the same, as would be expected for chimeric reads that result from library construction (Figure 6B). We further estimated the average number of *de novo P-element* insertions per genome (Figure 6C), and we arrived at ∼1 insertion per genome in *Har* flies and ∼3 insertions per genome in 2–4-day F1 flies, increasing to ∼8 per genome in 21-day F1 flies and falling back down to ∼4 insertions per genome in 21-day F2 flies. These estimates are consistent with the reestablishment of *P-element*-targeting piRNAs in hybrid-dysgenic flies (35).

Our previous study used TEMP and identified many new insertions for most of the residential transposon families in F1 and F2 flies, i.e. insertions not in their parents. Notably, *roo* appeared to be even more active than *P-element* (35). Roughly 40% of the new insertion reads detected by TEMP were singleton reads, which were likely chimeric reads, while the remaining 60% of new insertion reads formed 1p1 and 2p insertions and were unlikely chimeric reads (Supplementary Figure S6A). Using TEMP2, we largely reproduced the results on ‘new’ 1p1 and 2p insertions that were not in parental samples, including the high numbers of insertion reads for *roo* (Supplementary Figure S6B). TEMP2 verified that these 1p1 and 2p ‘new’ insertions are supported by multiple reads and hence are not likely derived from chimera. About half of the ‘new’ 1p1 and 2p insertions (46.3% and 49.3% in 2–4-day and 21-day F1 flies, respectively) are inserted in the same genomic positions between the two populations of F1 flies, suggesting that these insertions already existed in the parental flies but were undetected by the sequencing data (and TEMP2). These ‘new’ germline insertions are of low frequencies (median = 0.098 and 0.111 in the 2–4-day and 21-day *wH* flies, respectively), even though they are supported by multiple reads, and they are difficult to detect in parental strains. Furthermore, TEMP2 removed the singleton reads that were likely chimeric reads (Supplementary Figure S6B) and estimated much lower numbers of *de novo* insertions for residential transposons (one insertion per transposon family per genome) than for *P-element* (3–8 insertions per genome, Supplementary Figure S6C). Nevertheless, due to the large number of residential transposon families in the fly genome (over 100), these transposons can still exert a toll on the germline genome.

### Bias at transposon insertion sites detected in Illumina libraries

**Figure 7.**
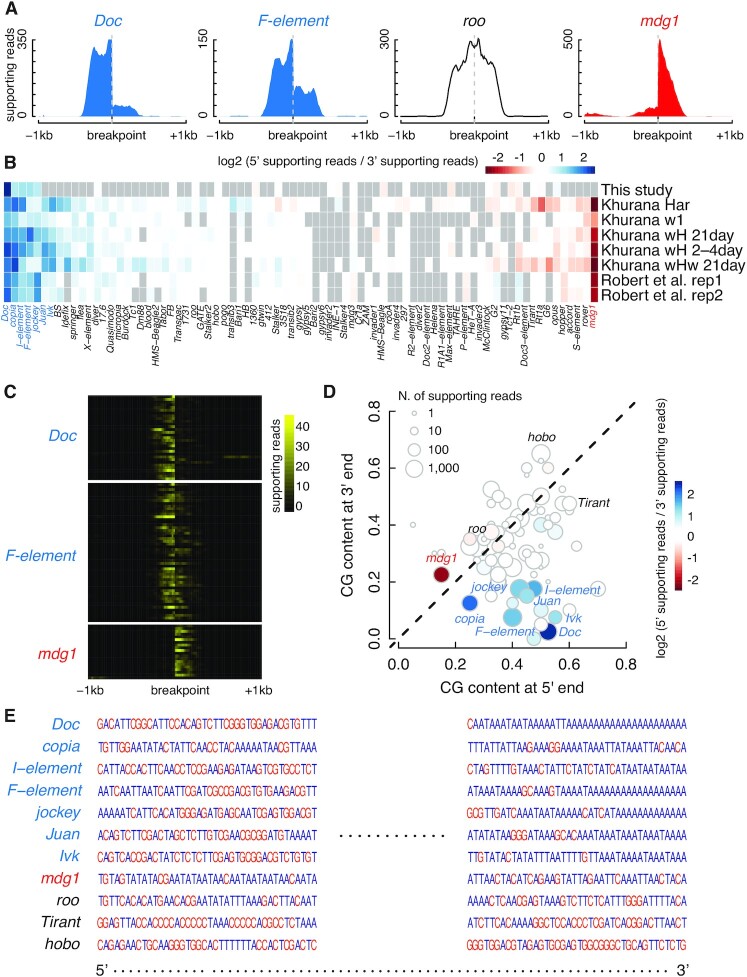
Sequence coverage bias at the genomic regions flanking inserted transposons. (**A**) Low read coverage at the 3′ flanking regions of *Doc* and *F-element* transposons and the 5′ flanking regions of the *mdg1* transposon at their insertion sites. Read coverage profiles in the ±1 kb window centered on the breakpoints of new *Doc*, *F-element*, *roo*, and *mdg1* insertions. *Doc* and *F-element* (in blue) show many more reads at its 5′ flanking genomic regions than its 3′ flanking genomic regions, while *mdg1* (in red) shows the opposite trend. Some transposons, like *roo* (in white), show no bias in read coverage. (**B**) Biases of the sequence coverage flanking inserted transposons. Heatmap depicts the sequence coverage bias for fly transposons of eight Illumina datasets from three different sources. Transposons with at least one germline insertion detected in at least one of the eight datasets were included, 57 transposons in total. The blue to red color spectrum indicates 5′ to 3′ coverage bias, with white corresponding to no bias. Transposons that do not have sufficient supporting reads in a particular dataset are in gray. (**C**) Bias at the 3′-ends of *Doc* and *F-element* insertions and the 5′-end of *mdg1* insertions. Heatmaps depict the read coverage in the flanking regions of *Doc, F-element*, and *mdg1* insertions, with each row indicating one insertion. All 31 *Doc* insertions, 51 *F-element* insertions, and 20 *mdg1* insertions in the *Har* dataset are included. (**D**) The sequence coverage bias flanking inserted transposons is correlated with the CG content at the transposons’ two ends. Scatterplot compares the CG content of the first and last 40 nucleotides for each of the 57 transposons in B, colored accordingly by sequence coverage bias. (**E**) Two ends of several example transposons reveal their different CG contents. The first and last 40 nucleotides of the consensus sequences of seven 5′-biased transposons, one 3′-biased transposon and three unbiased transposons. Cs and Gs are in orange while As and Ts are in dark blue. Poly-ATs are enriched at the 3′-ends of *Doc*, *Copia*, *I-element*, *F-element*, *jockey*, *Juan* and *Ivk*, but at the 5′-end of *mdg1*.

When we compared Illumina and PacBio data for detecting germline transposon insertions (Figure 4), we noticed that some transposon insertions were supported by different numbers of Illumina reads at the two ends (Figure 7A), but PacBio reads around these transposon insertions were evenly distributed. To investigate the potential bias of Illumina library construction and sequencing, we compared 5′-supporting reads and 3′-supporting reads for each transposon insertion that was supported by five or more reads in at least one of the dysgenic-hybrid fly datasets (35). We consistently observed more 5′-supporting reads than 3′-supporting reads for some transposon families (e.g. *Doc* and *F-element*) but more 5′-supporting reads than 3′-supporting reads for other transposon families (e.g. *mdg1*), although most transposon families (e.g. *roo*) have similar numbers of 5′- and 3′-supporting reads (Figure 7A, B), and this bias is consistently observed at individual insertion sites (Figure 7C). We analyzed two more Illumina datasets from an independent study (36), and the results confirmed these patterns (Figure 7B).

Five of seven transposon families with biased supporting reads (*Doc*, *I-element*, *F-element*, *jockey*, and *Juan*) belong to LINE elements, which have AT-rich 3′-ends (37,38). This observation motivated us to compute the CG content (percentage of C and G nucleotides) at the two ends of the transposon families and, indeed, the families with the most bias toward 5′-supporting reads tend to have the highest GC-content at the 5′-end while lowest GC-content at the 3′-end (Figure 7D, E). Interestingly, there are few transposons with higher GC-content at their 3′-end than their 5′-end. *mdg1*, which has a 3′-end bias in supporting reads (Figure 7A), has low GC-content at both ends, although slightly lower at its 5′-end than its 3′-end. The imbalance in supporting reads could result from library construction or Illumina sequencing; previous studies have reported dependence of sequencing coverage on CG contents (39–41). The imbalance could affect the accuracy of estimating insertion frequencies and even insertion detection when the read depth is low.

## DISCUSSION

Transposons are one of the major forces driving the evolution of Metazoan genomes. However, the identification of new transposon insertions remains a challenge due to the repetitive nature of their sequences and the imperfectness of genome assemblies. Although long-read sequencing techniques such as PacBio can produce reads that are long enough to span an entire transposon insertion, such techniques are costly and do not produce sufficient numbers of reads for estimating insertion frequencies or detection of *de novo* insertions. The rapid accumulation of short-read whole-genome sequencing has provided ample opportunities and challenges for computational algorithms designed to detect transposon insertions in a wide variety of samples. Responding to these opportunities and challenges, we performed PacBio and Illumina sequencing on *Drosophila* and used the data to build a benchmark of transposon insertions, which can be used as a benchmark to test transposon detection algorithms. The long reads of our PacBio sequencing data can also be used by future studies for predicting complex transposon insertions in *Drosophila melanogaster*.

We further present a designed TEMP2 algorithm, which can accurately identify germline insertions, estimate their frequencies, and pinpoint their breakpoints. We compared TEMP2 with several state-of-the-art algorithms using simulated data, our benchmark in *Drosophila*, and a set of human transposons curated by the 1000 Genomes Project, and TEMP2 performed competitively in all these comparisons. Furthermore, TEMP2 has the newly developed capability of estimating the level of low-frequency transposon insertions generated from recent activity, which can be applied to distinguish inherited from *de novo* transposon insertions.

There are several challenges for identifying transposon insertions, and we designed TEMP2 to tackle these challenges. First, it is difficult to identify transposon insertions into repetitive regions of the genome, especially regions that already have an annotated insertion of the same transposon or of a transposon in the same family. The sequence mapping algorithm bwa mem identifies many ‘discordant’ reads at such regions, yet these reads likely originate from somewhere else in the genome with another copy of the transposon. We thus filter out all transposon insertions that likely result from such alignment artifacts. Second, we examine the overlapping insertions of different transposons in a region and only retain the insertions with the most supporting reads. This is again designed to guard against alignment artifacts in highly repetitive regions. This is why MELT performs well in the human dataset but poorly in the *Drosophila* dataset because MELT does not distinguish homologous transposons. In the human data, the three assessed transposons, LINE1, SVA and ALU, are highly divergent; however, in flies, many of the transposons are homologous, and these homologous transposons introduced a lot of false-positive insertions when MELT was used for transposon detection. Third, chimeric reads are prevalent in short-read sequencing libraries. To minimize false positives caused by chimeric reads in identifying *de novo* transposon insertions, we take advantage of the fact that *bona fide* transposon-supporting reads must map to the two ends of most transposons and cannot reach the transposon interior, due to short fragment length. Third, the identification of short transposons poses unique challenges. For example, a full-length Alu element is 280-nt long, which is shorter than the fragment length of the high-depth Illumina libraries (mean = 700 nt, estimated by fully mapped reads). As a result, an Alu element could be contained in a read-pair. TEMP2 combines clipped and unclipped discordant reads in the same cluster to detect insertions of short transposons.

The results of our simulated data indicate that sequencing depths higher than 10× genome coverage are sufficient for most algorithms, and TEMP2 performs well even at 5× genome coverage (Figure 2). Indeed, TEMP2’s performance on the 5.7× human dataset was comparable with the 60× human dataset, although its sensitivity decreased when the 60× dataset was downsampled to 14× (Supplementary Figure S5). A higher sequencing depth typically leads to a higher sensitivity but for some algorithms leads to a lower precision, although this does not seem to be the case for TEMP2 (Figure 2; comparing Figure 4 with Supplementary Figure S5). Thus, we recommend a sequencing depth in the 10–30× range as a cost-effective middle ground.

Many algorithms use unbalanced 5′ and 3′ supporting reads as a criterion for filtering out insertion predictions, e.g. TEMP, ERVcaller and MELT (10,26,27). However, we found that some *Drosophila* transposons have highly unbalanced supporting reads at their two ends. This may be caused by the differential CG contents at the two ends of a transposon. For example, the LINE family of *Drosophila* transposons tend to have low CG content at their 3′-ends, which correspond to fewer supporting reads. Therefore, a filtering criterion based on unbalanced supporting reads would miss real insertions for such transposons.

Our analysis of hybrid dysgenic fly data confirmed the active transposition of *P-element* in F1 flies resulting from the cross between *Har* males, which have *P-elements* in their genome, and *w^1^* females, which don’t. We estimated that, due to the lack of maternal piRNAs targeting *P-elements*, F1 flies gained about 3 new *P-element* insertions per genome in their first 2–4 days and about five new *P-element* insertions per genome in their first 21 days, when piRNAs targeting *P-elements* are regenerated. In the F2 generation, when the piRNAs targeting *P-elements* have been replenished, the new *P-element* insertions went back down to three per genome (Figure 5). This new capability of TEMP2 for analyzing transposon movement can be applied to other data involving mutants that derepress transposons and infections by viruses that integrate them into the host genome (42).

TEMP2 has two limitations in estimating *de novo* transposon insertions. First, TEMP2 cannot distinguish low-frequency germline insertions from *de novo* insertions, when it is used to analyze a pool of genomes. For sequencing data on individual genomes, such as human or mouse, the frequencies of germline insertions are either 0.5 or 1 with the exception of the genomic regions with copy number variations. However, in pooled sequencing where the genomes of multiple individuals are included in the library construction, such as the fly ovary data we analyzed, low frequent transpositions can be misassigned as somatic transpositions, especially when the read coverage is low. With the rapid improvement of single-cell sequencing technologies, we hope to integrate single-cell data with bulk data to better characterize transposon insertions in future work (43). Second, TEMP2 cannot estimate the *de novo* insertion rate for short transposons such as Alu elements. TEMP2 needs center-mapping reads to estimate the false-discovery rate of transposon-supporting singleton reads; however, it is not possible to define the center for transposons whose lengths are shorter than the fragment lengths of the sequencing library.

In summary, TEMP2 is a new method for identifying transposon insertions in short-read genomic DNA sequencing data. It combines the good features of existing algorithms and adds new features for eliminating false-positive predictions while maintaining high sensitivity. It performs stably in low sequencing depth and varying read length and works well for all sizes of transposons. TEMP2 was tested on both simulated and experimental data and showed overall better performance in a number of metrics than start-of-the-art methods. Its new capability of detecting *de novo* transposon insertions should find novel uses in studying the movements of active mobile genomic elements.

## DATA AVAILABILITY

All simulated data were deposited in https://publications.wenglab.org/TEMP2/download/. TEMP2 is freely available at Github: https://github.com/weng-lab/TEMP2. All deep-sequencing data generated in this study were deposited in the Sequence Read Archive (SRA) under accession number PRJNA636174.

## Supplementary Material

gkab010_Supplemental_Files

## References

[1] Huang C.R.L. , Burns K.H., Boeke J.D Active transposition in genomes. Annu. Rev. Genet. 2012; 46:651–675. 23145912 10.1146/annurev-genet-110711-155616 PMC3612533

[2] Britten R.J. Transposable element insertions have strongly affected human evolution. Proc. Natl. Acad. Sci. U.S.A. 2010; 107:19945–19948. 21041622 10.1073/pnas.1014330107 PMC2993358

[3] Hedges D.J. , Belancio V.P. Restless genomes humans as a model organism for understanding host-retrotransposable element dynamics. Adv. Genet. 2011; 73:219–262. 21310298 10.1016/B978-0-12-380860-8.00006-9 PMC4433537

[4] Bennetzen J.L. Transposable element contributions to plant gene and genome evolution. Plant Mol. Biol. 2000; 42:251–269. 10688140

[5] Belancio V.P. , Hedges D.J., Deininger P. Mammalian non-LTR retrotransposons: for better or worse, in sickness and in health. Genome Res. 2008; 18:343–358. 18256243 10.1101/gr.5558208

[6] Iskow R.C. , McCabe M.T., Mills R.E., Torene S., Pittard W.S., Neuwald A.F., Van Meir E.G., Vertino P.M., Devine S.E. Natural mutagenesis of human genomes by endogenous retrotransposons. Cell. 2010; 141:1253–1261. 20603005 10.1016/j.cell.2010.05.020 PMC2943760

[7] Shukla R. , Upton K.R., Muñoz-Lopez M., Gerhardt D.J., Fisher M.E., Nguyen T., Brennan P.M., Baillie J.K., Collino A., Ghisletti S. et al. Endogenous retrotransposition activates oncogenic pathways in hepatocellular carcinoma. Cell. 2013; 153:101–111. 23540693 10.1016/j.cell.2013.02.032 PMC3898742

[8] Solyom S. , Kazazian H.H. Jr Mobile elements in the human genome: implications for disease. Genome Med. 2012; 4:12. 22364178 10.1186/gm311 PMC3392758

[9] Treiber C.D. , Waddell S. Resolving the prevalence of somatic transposition in Drosophila. eLife. 2017; 6:e28297. 28742021 10.7554/eLife.28297 PMC5553932

[10] Gardner E.J. , Lam V.K., Harris D.N., Chuang N.T., Scott E.C., Pittard W.S., Mills R.E. 1000 Genomes Project Consortium and Devine, S.E. The mobile element Locator tool (MELT): population-scale mobile element discovery and biology. Genome Res. 2017; 27:1916–1929. 28855259 10.1101/gr.218032.116 PMC5668948

[11] Keane T.M. , Wong K., Adams D.J. RetroSeq: transposable element discovery from next-generation sequencing data. Bioinformatics. 2013; 29:389–390. 23233656 10.1093/bioinformatics/bts697 PMC3562067

[12] Chen J. , Wrightsman T.R., Wessler S.R., Stajich J.E. RelocaTE2: a high resolution transposable element insertion site mapping tool for population resequencing. PeerJ. 2017; 5:e2942. 28149701 10.7717/peerj.2942 PMC5274521

[13] Zhuang J. , Wang J., Theurkauf W., Weng Z. TEMP: a computational method for analyzing transposable element polymorphism in populations. Nucleic Acids Res. 2014; 42:6826–6838. 24753423 10.1093/nar/gku323 PMC4066757

[14] Chen X. , Li D ERVcaller: identifying polymorphic endogenous retrovirus and other transposable element insertions using whole-genome sequencing data. Bioinformatics. 2019; 35:3913–3922. 30895294 10.1093/bioinformatics/btz205

[15] Goerner-Potvin P. , Bourque G. Computational tools to unmask transposable elements. Nat. Rev. Genet. 2018; 19:688–704. 30232369 10.1038/s41576-018-0050-x

[16] Li H. , Durbin R. Fast and accurate short read alignment with Burrows-Wheeler transform. Bioinformatics. 2009; 25:1754–1760. 19451168 10.1093/bioinformatics/btp324 PMC2705234

[17] Tang Z. , Steranka J.P., Ma S., Grivainis M., Rodić N., Huang C.R.L., Shih I.-M., Wang T.-L., Boeke J.D., Fenyö D. et al. Human transposon insertion profiling: Analysis, visualization and identification of somatic LINE-1 insertions in ovarian cancer. Proc. Natl. Acad. Sci. U.S.A. 2017; 114:E733–E740. 28096347 10.1073/pnas.1619797114 PMC5293032

[18] Burns K.H. , Boeke J.D. Human transposon tectonics. Cell. 2012; 149:740–752. 22579280 10.1016/j.cell.2012.04.019 PMC3370394

[19] Huang W. , Li L., Myers J.R., Marth G.T. ART: a next-generation sequencing read simulator. Bioinformatics. 2012; 28:593–594. 22199392 10.1093/bioinformatics/btr708 PMC3278762

[20] Li H. Minimap2: pairwise alignment for nucleotide sequences. Bioinformatics. 2018; 34:3094–3100. 29750242 10.1093/bioinformatics/bty191 PMC6137996

[21] Sedlazeck F.J. , Rescheneder P., Smolka M., Fang H., Nattestad M., von Haeseler A., Schatz M.C. Accurate detection of complex structural variations using single-molecule sequencing. Nat. Methods. 2018; 15:461–468. 29713083 10.1038/s41592-018-0001-7 PMC5990442

[22] Robinson J.T. , Thorvaldsdóttir H., Winckler W., Guttman M., Lander E.S., Getz G., Mesirov J.P. Integrative genomics viewer. Nat. Biotechnol. 2011; 29:24–26. 21221095 10.1038/nbt.1754 PMC3346182

[23] Thurmond J. , Goodman J.L., Strelets V.B., Attrill H., Gramates L.S., Marygold S.J., Matthews B.B., Millburn G., Antonazzo G., Trovisco V. et al. FlyBase 2.0: the next generation. Nucleic Acids Res. 2019; 47:D759–D765. 30364959 10.1093/nar/gky1003 PMC6323960

[24] Smit A.F.A. , Hubley R., Green P. RepeatMasker Open-4.0. 2013-2015; http://www.repeatmasker.org.

[25] Li H. , Handsaker B., Wysoker A., Fennell T., Ruan J., Homer N., Marth G., Abecasis G., Durbin R. 1000 Genome Project Data Processing Subgroup The Sequence Alignment/Map format and SAMtools. Bioinformatics. 2009; 25:2078–2079. 19505943 10.1093/bioinformatics/btp352 PMC2723002

[26] Zhuang J. , Wang J., Theurkauf W., Weng Z. TEMP: a computational method for analyzing transposable element polymorphism in populations. Nucleic Acids Res. 2014; 42:6826–6838. 24753423 10.1093/nar/gku323 PMC4066757

[27] Chen X. , Li D ERVcaller: identifying polymorphic endogenous retrovirus and other transposable element insertions using whole-genome sequencing data. Bioinformatics. 2019; 35:3913–3922. 30895294 10.1093/bioinformatics/btz205

[28] Keane T.M. , Wong K., Adams D.J. RetroSeq: transposable element discovery from next-generation sequencing data. Bioinformatics. 2013; 29:389–390. 23233656 10.1093/bioinformatics/bts697 PMC3562067

[29] Chen J. , Wrightsman T.R., Wessler S.R., Stajich J.E. RelocaTE2: a high resolution transposable element insertion site mapping tool for population resequencing. PeerJ. 2017; 5:e2942. 28149701 10.7717/peerj.2942 PMC5274521

[30] Hoskins R.A. , Carlson J.W., Wan K.H., Park S., Mendez I., Galle S.E., Booth B.W., Pfeiffer B.D., George R.A., Svirskas R. et al. The Release 6 reference sequence of the Drosophila melanogaster genome. Genome Res. 2015; 25:445–458. 25589440 10.1101/gr.185579.114 PMC4352887

[31] dos Santos G. , Schroeder A.J., Goodman J.L., Strelets V.B., Crosby M.A., Thurmond J., Emmert D.B., Gelbart W.M. FlyBase Consortium FlyBase: introduction of the Drosophila melanogaster Release 6 reference genome assembly and large-scale migration of genome annotations. Nucleic Acids Res. 2015; 43:D690–D697. 25398896 10.1093/nar/gku1099 PMC4383921

[32] Rishishwar L. , Mariño-Ramírez L., Jordan I.K. Benchmarking computational tools for polymorphic transposable element detection. Brief. Bioinform. 2017; 18:908–918. 27524380 10.1093/bib/bbw072 PMC5808724

[33] Kriegs J.O. , Churakov G., Jurka J., Brosius J., Schmitz J. Evolutionary history of 7SL RNA-derived SINEs in supraprimates. Trends Genet. 2007; 23:158–161. 17307271 10.1016/j.tig.2007.02.002

[34] Deininger P.L. , Moran J.V., Batzer M.A., Kazazian H.H. Jr Mobile elements and mammalian genome evolution. Curr. Opin. Genet. Dev. 2003; 13:651–658. 14638329 10.1016/j.gde.2003.10.013

[35] Khurana J.S. , Wang J., Xu J., Koppetsch B.S., Thomson T.C., Nowosielska A., Li C., Zamore P.D., Weng Z., Theurkauf W.E. Adaptation to P element transposon invasion in Drosophila melanogaster. Cell. 2011; 147:1551–1563. 22196730 10.1016/j.cell.2011.11.042 PMC3246748

[36] Armstrong R.L. , Penke T.J.R., Chao S.K., Gentile G.M., Strahl B.D., Matera A.G., McKay D.J., Duronio R.J H3K9 promotes under-replication of pericentromeric heterochromatin in drosophila salivary gland polytene chromosomes. Genes. 2019; 10:93. 10.3390/genes10020093 PMC6409945 30700014

[37] Kajikawa M. , Okada N. LINEs mobilize SINEs in the Eel through a shared 3′ sequence. Cell. 2002; 111:433–444. 12419252 10.1016/s0092-8674(02)01041-3

[38] Chambeyron S. , Bucheton A., Busseau I. Tandem UAA repeats at the 3′-End of the transcript are essential for the precise initiation of reverse transcription of the I factor indrosophila melanogaster. J. Biol. Chem. 2002; 277:17877–17882. 11882661 10.1074/jbc.M200996200

[39] Sims D. , Sudbery I., Ilott N.E., Heger A., Ponting C.P. Sequencing depth and coverage: key considerations in genomic analyses. Nat. Rev. Genet. 2014; 15:121–132. 24434847 10.1038/nrg3642

[40] Nakamura K. , Oshima T., Morimoto T., Ikeda S., Yoshikawa H., Shiwa Y., Ishikawa S., Linak M.C., Hirai A., Takahashi H. et al. Sequence-specific error profile of Illumina sequencers. Nucleic Acids Res. 2011; 39:e90. 21576222 10.1093/nar/gkr344 PMC3141275

[41] Benjamini Y. , Speed T.P. Summarizing and correcting the GC content bias in high-throughput sequencing. Nucleic Acids Res. 2012; 40:e72. 22323520 10.1093/nar/gks001 PMC3378858

[42] Yu T. , Koppetsch B.S., Pagliarani S., Johnston S., Silverstein N.J., Luban J., Chappell K., Weng Z., Theurkauf W.E. The piRNA response to retroviral invasion of the Koala genome. Cell. 2019; 179:632–643. 31607510 10.1016/j.cell.2019.09.002 PMC6800666

[43] Evrony G.D. , Lee E., Park P.J., Walsh C.A. Resolving rates of mutation in the brain using single-neuron genomics. Elife. 2016; 5:e12966. 26901440 10.7554/eLife.12966 PMC4805530

