# Supplementary material for: A benchmark and an algorithm for detecting germline transposon insertions and measuring de novo transposon insertion frequencies
Source: Nucleic Acids Res. 2021 Jan 28;49(8):e44. doi: 10.1093/nar/gkab010 (PMC8096211; doi:10.1093/nar/gkab010)
Supplement: gkab010_Supplemental_Files [file gkab010_supplemental_files.zip › TEMP2 (Supplementary Materials).pdf]

# Supplementary Figures

## A Benchmark and An Algorithm for Detecting Germline Transposon Insertions and Measuring *de novo* Transposon Insertion Frequencies

Tianxiong Yu[1,2], Xiao Huang[1], Shengqian Dou[3], Xiaolu Tang[3], Shiqi Luo[3], William E. Theurkauf[4*], Jian Lu[3*], and Zhiping Weng[1,2*]

[1]Department of Thoracic Surgery, Clinical Translational Research Center, Shanghai Pulmonary Hospital, The School of Life Sciences and Technology, Tongji University, Shanghai 200092, China
[2]Program in Bioinformatics and Integrative Biology, University of Massachusetts Medical School, Worcester, Massachusetts, United States of America
[3]State Key Laboratory of Protein and Plant Gene Research, Center for Bioinformatics, School of Life Sciences and Peking-Tsinghua Center for Life Sciences, Peking University, Beijing 100871, China
[4]Program in Molecular Medicine, University of Massachusetts Medical School, Worcester, MA 01605, USA
[*]Corresponding authors

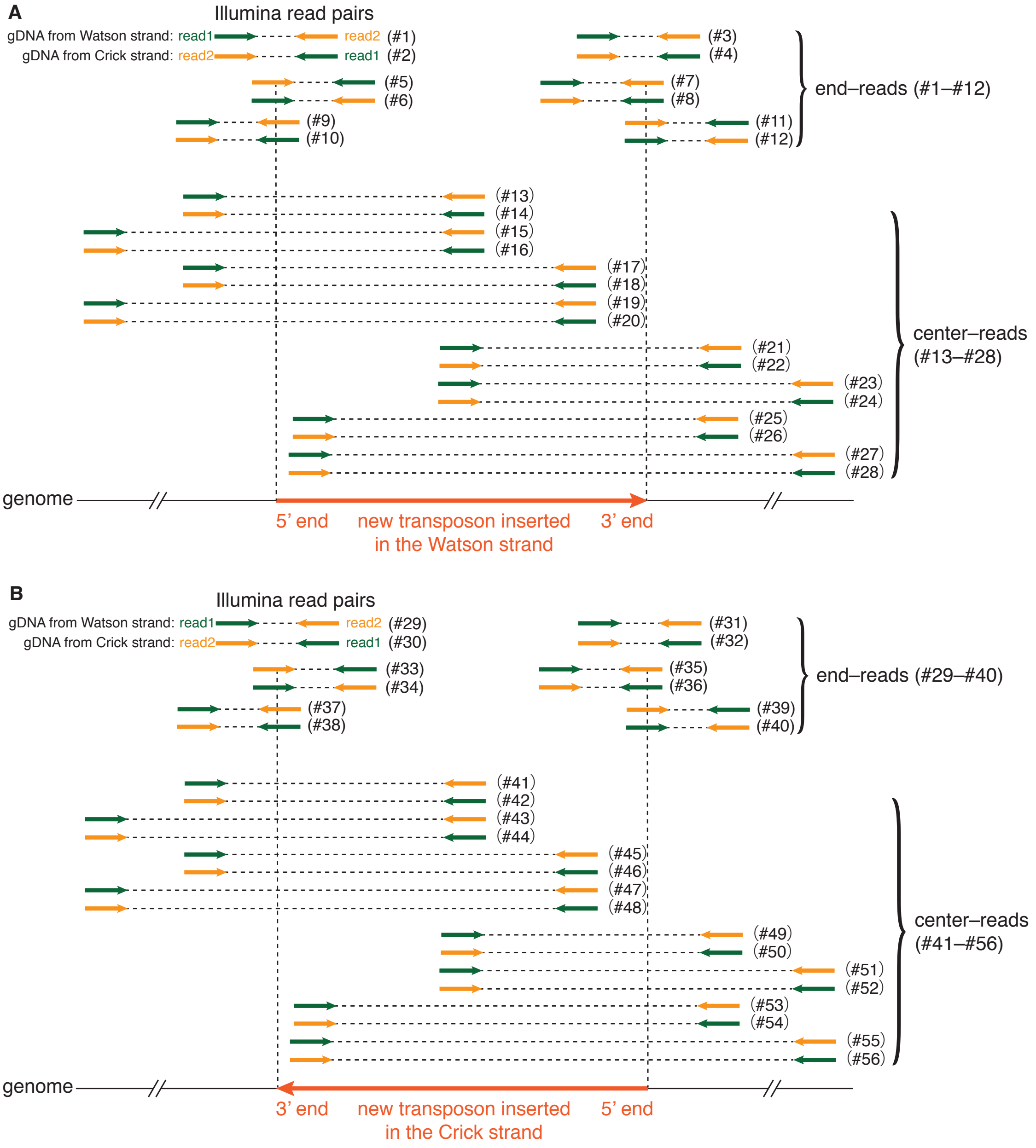


**Supplementary Figure S1 (Related to Figure 1). A diagram depicts the details of how TEMP2 processes discordant and spit reads.**
A transposon is inserted into the Watson strand (panel **A**) or Crick strand (panel **B**) of the genome. Discordant and split reads are numbered from #1 to #56. TEMP2 regards the reads that map to the proper strand of the two ends of the transposon consensus sequence as end-reads and the remaining reads as center-reads for measuring the *de novo* insertion rate. Because the two reads from a fragment are always sequenced convergely, the end-reads that map to the 5′-end of a transposon consensus sequence should be antisense to this transposon and the end-reads that map to the 3′-end of the transposon consensus sequence should be sense to this transposon, regardless whether this transposon is inserted into the Watson strand or the Crick strand of the genome. Read1 in a read-pair is colored in green while read2 is colored in yellow.

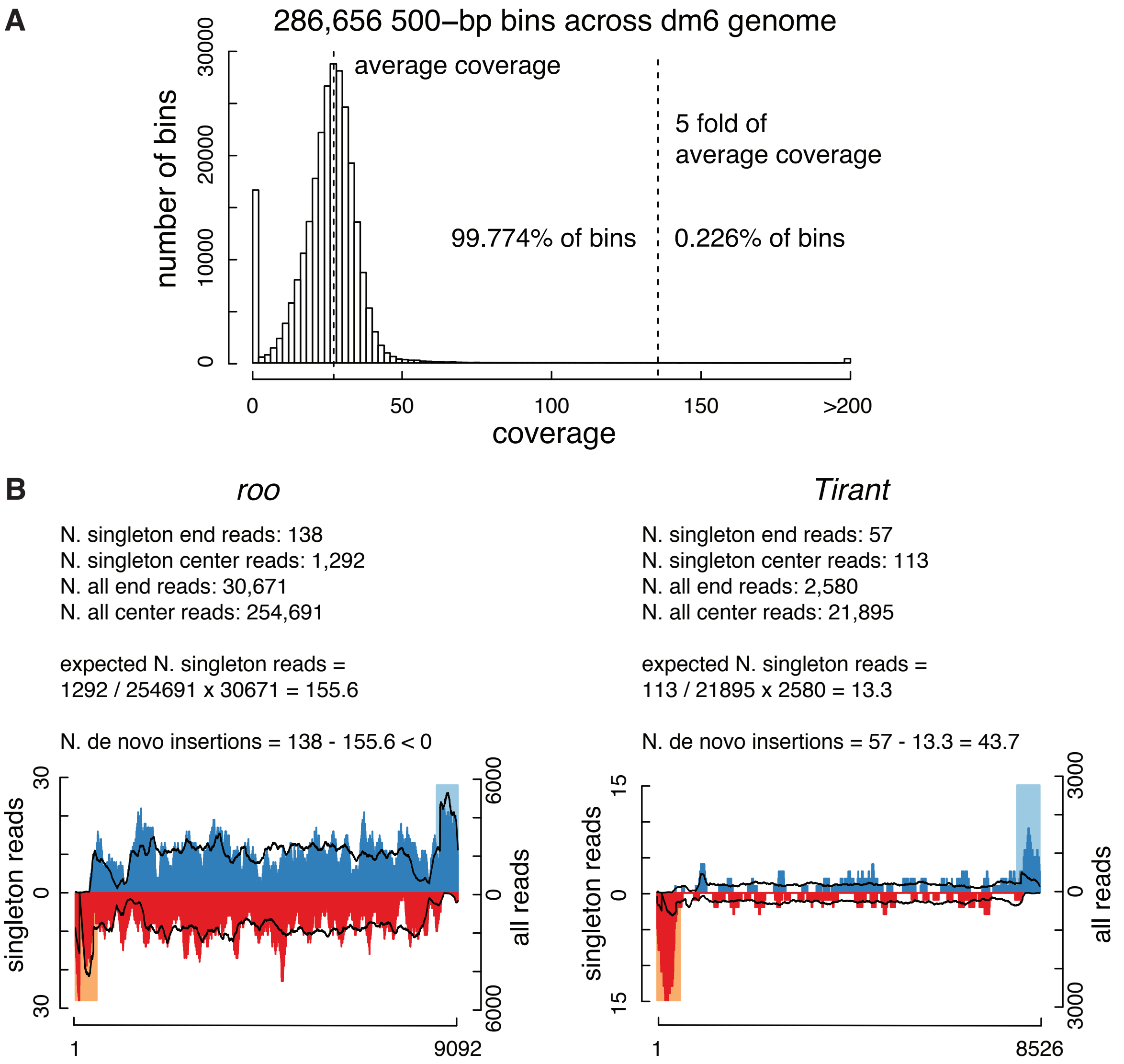


**Supplementary Figure S2 (Related to Figure 1). A histogram shows how TEMP2 determines the genome coverage threshold and two examples illustrate how TEMP2 estimates de novo insertion frequencies.**

**A.** Histogram shows the coverage of 286,656 500-bp bins across the fly genome (dm6). The average coverage and 5 times the average coverage are marked by two vertical dashed lines. 99.774% of these bins have less than 5 times the average coverage and the rest bins are filtered out by TEMP2 in the process of detecting new transposon insertions.

**B.** Two examples of estimating the rate of *de novo* transposon insertions. Using the left Y-axis, singleton reads aligned to the sense strands of the transposon consensus sequences are tallied to make the blue curve (including read-pairs #3, #4, #7, #8, #11, #12, #21–#30, #33, #34, #37, #38, and #41–#48 in **Supplementary Figure S2**) while singleton reads aligned to the antisense strands of transposon consensus sequences are tallied to make the red curve (including read-pairs #1, #2, #5, #6, #9, #10, #13–#20, #31, #32, #35, #36, #39, #40, and #49–#56 in **Supplementary Figure S2**). The two ends of each transposon are highlighted in light blue and light orange. Using the right Y-axis, two black curves indicate all raw reads mapped to the transposon consensus sequence, which were used to perform normalization while estimating the *de novo* transposon insertion rates.

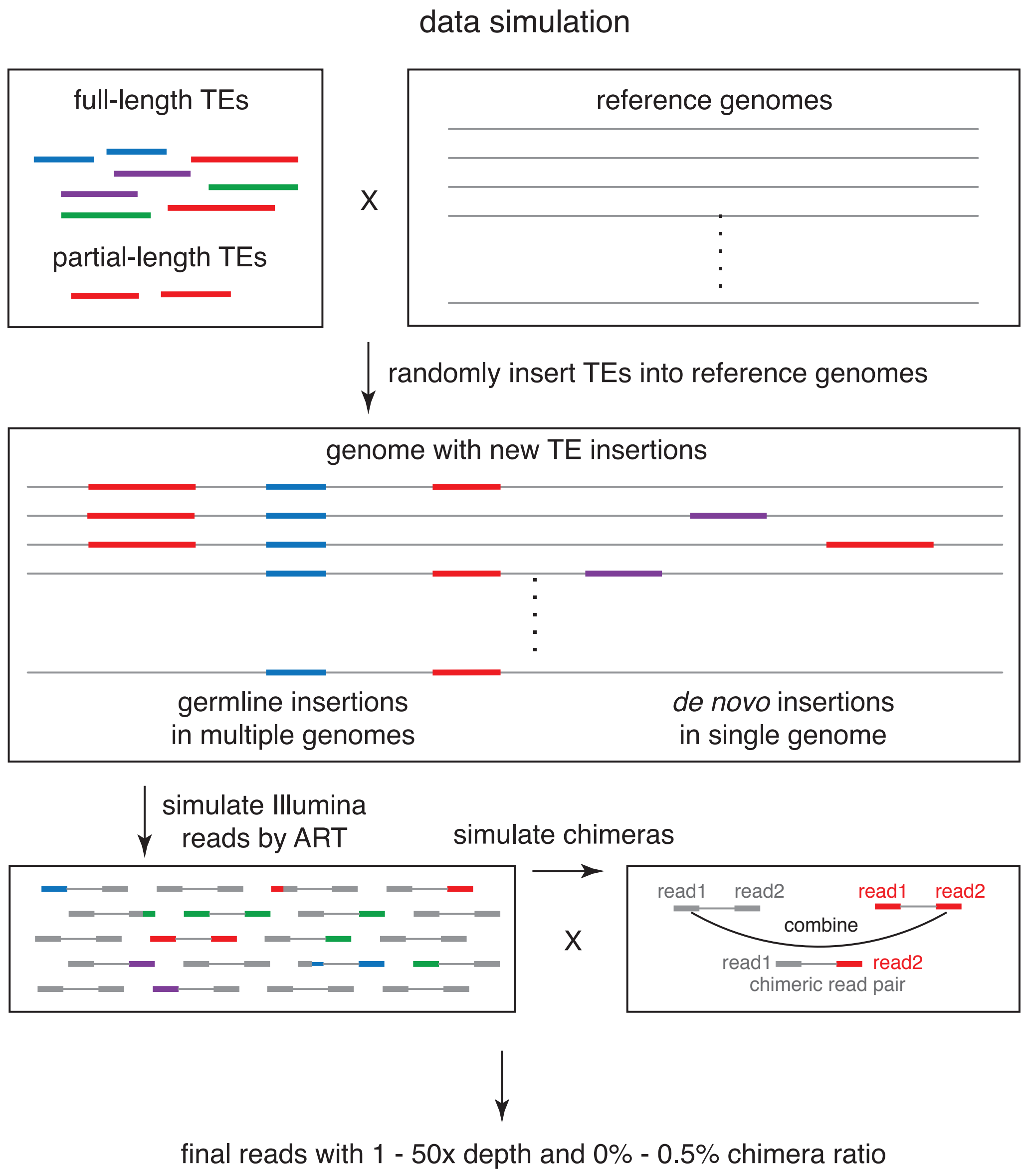


**Supplementary Figure S3 (Related to Figure 2). A summary diagram depicts how the simulated data were generated.**
We performed the simulation in two steps: genome simulation and read-pair simulation. The reference genome is represented as a thin grey line while different transposon subfamilies are depicted as short colored lines. Paired-end reads are depicted as pairs of boxes each connected by a short horizontal line. The portion of a read mapping to the reference genome is in grey, while the portion of the read mapping to a transposon is in color. A chimeric read-pair was generated by randomly connecting the first read of one read-pair and the second read of another read-pair. We generated multiple datasets of 100-bp paired-end (PE100) reads with 0–0.5% chimeric read-pairs and 1–50X sequencing depths.

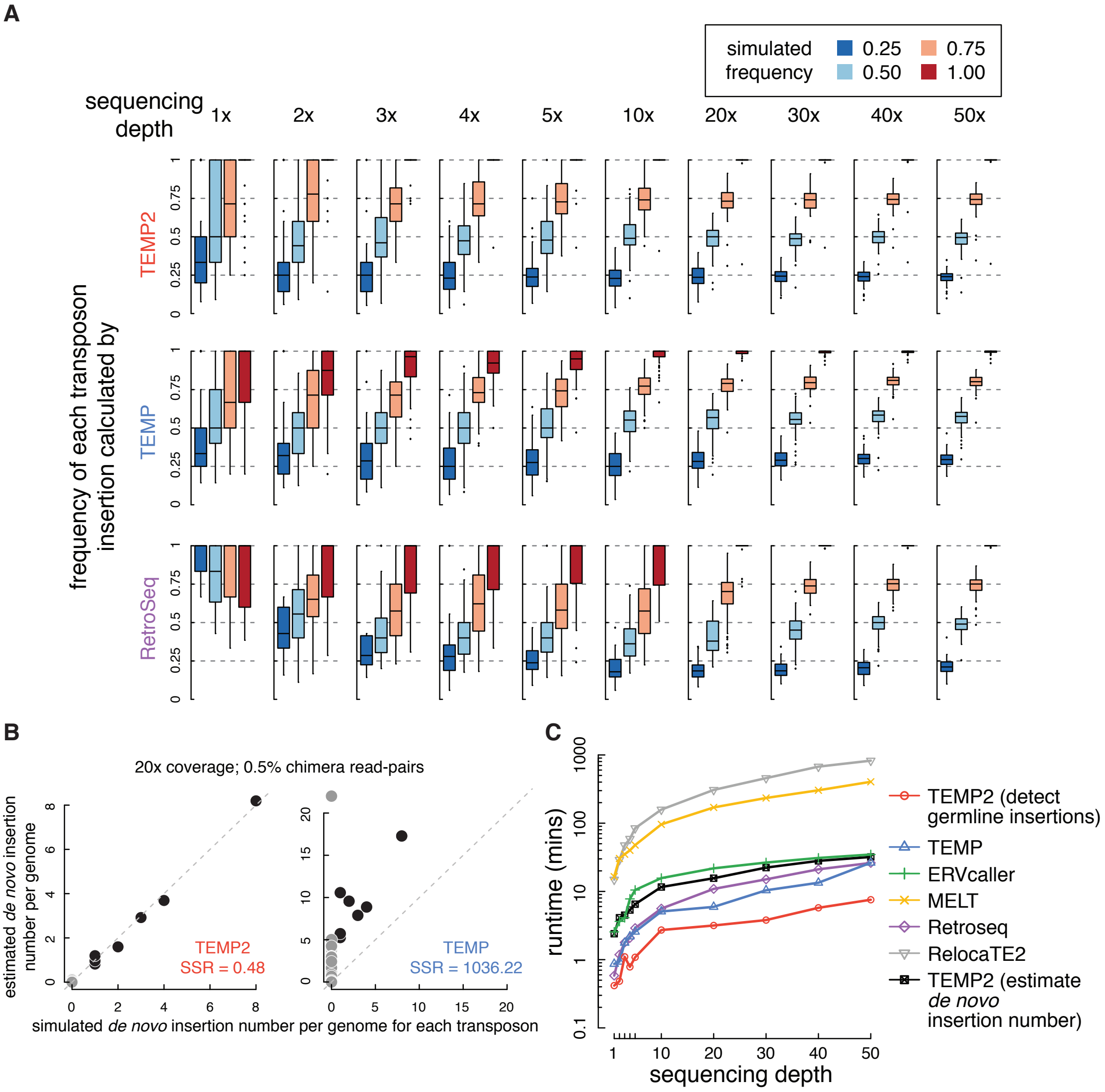


**Supplementary Figure S4 (Related to Figure 2). TEMP2 runs faster and estimates the frequencies of transposon insertions more accurately than other algorithms.**
**A.** Box plots show the frequencies of germline transposon insertions predicted by TEMP2, TEMP, and RetroSeq. Simulated Illumina read-pairs at different sequencing depths (0-50X genome coverage) with 0.05% chimera reads were used for the comparison. The simulated transposon insertions are separated into four groups based on their insertion frequencies (0.25, 0.5, 0.75 and 1), colored blue, light blue, light orange, and red, respectively. Horizontal dashed lines indicate the simulated frequencies—0.25, 0.5, 0.75 and 1. All boxplots in this Manuscript use the default definition, whereby (1) boxes represent the first and third quartiles; (2) the horizontal midline is the median, and (3) whiskers show the maximum and minimum values excluding outliers. Outliers are defined as data points with values higher than the third quartile + 1.5 × interquartile range (IQR) or lower than the first quartile − 1.5 × IQR.  IQR is the third quartile minus the first quartile.
**B.** Corresponding to Figure 2F, but in a simulated dataset with 0.5% chimera read-pairs.
**C.** Line plot compares the runtime of transposon-detecting algorithms in simulated dataset with different sequencing depth (1–50X of the *Drosophila* genome) and 0.05% chimera read-pairs.

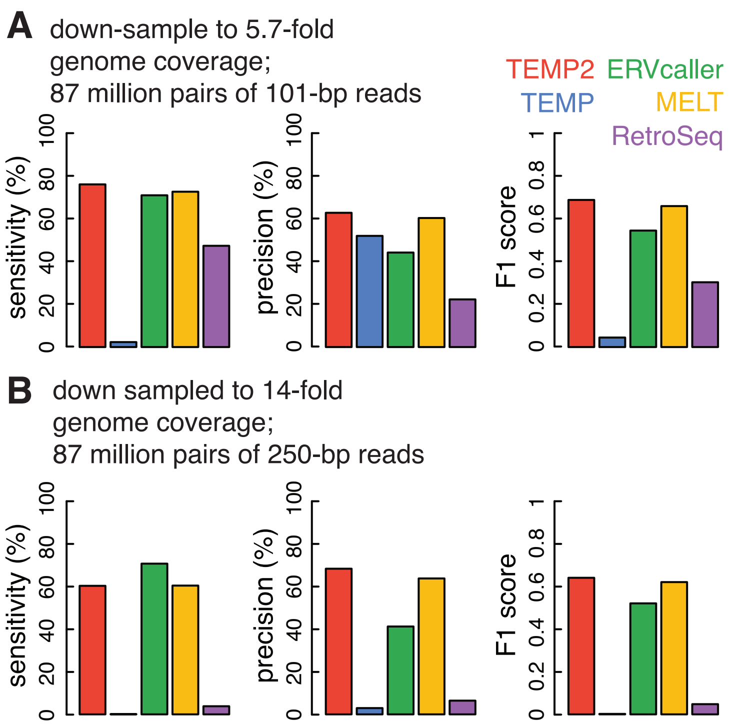


**Supplementary Figure S5 (Related to Figure 5). TEMP and RetroSeq have difficulty detecting short transposon insertions when sequencing reads are long.**
Corresponding to Figure 5, but the high-depth dataset in human NA12878 cells was downsampled to 5.7-fold genome coverage with reads clipped from the 3′-end to 101-nt long (**A**) or downsampled but not clipped (**B**; 14-fold genome coverage; 250-nt reads), and the performance of TEMP2, TEMP, ERVcaller, MELT and RetroSeq was evaluated.

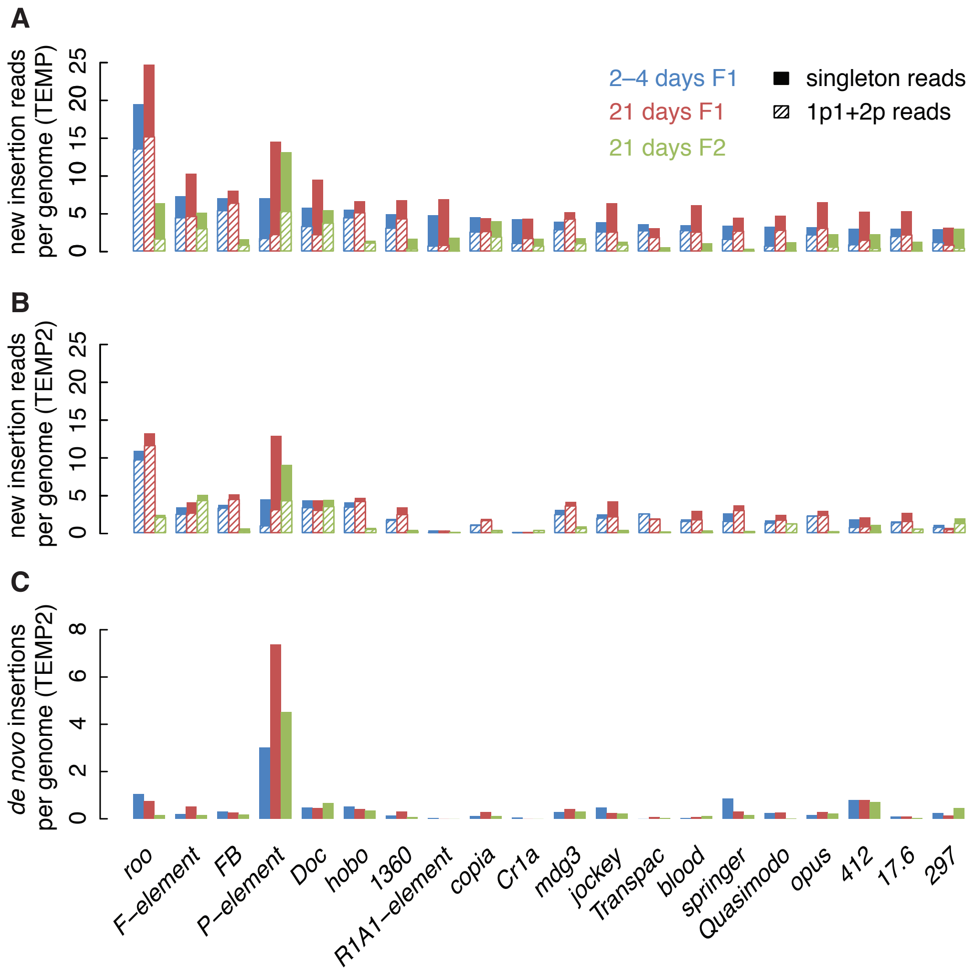


**Supplementary Figure S6 (Related to Figure 6). Reanalysis of the fly dysgenic dataset using TEMP2.**

New transposon insertions were detected by TEMP (top panel), TEMP2 (middle and bottom panels) following normalization of all three sequencing datasets to 18.3-fold genome coverage. (top and middle panels) Bars indicate normalized paired-end reads associated with new transposon insertions for specific transposon families in 2- to 4-day-old (blue bars) and 21-day-old dysgenic ovaries (red bars), relative to the parental Har and $w^1$ genomes. Green bars indicate new insertions in the fertile progeny of 21-day dysgenic females mated to the parental $w^1$ strain, and these insertions are relative to the 21-day-old dysgenic females. Hatched portions of the bars indicate new insertions with 1p1 and 2p reads, while solid portions indicate singleton reads. (bottom panel) Bars indicate the number of de novo insertions of each transposon family per genome detected by TEMP2. There are 9.8 new *P-element* singleton insertions in 21-day F1 flies that were not in parental flies. A lower number of 7.4 was estimated by the TEMP2 de novo insertion module, because TEMP2 subtracted out the predicted number of chimeric read-pairs.
